# Noise-Induced “Toughening” Effect in Wistar Rats: Enhanced Auditory Brainstem Responses Are Related to Calretinin and Nitric Oxide Synthase Upregulation

**DOI:** 10.3389/fnana.2016.00019

**Published:** 2016-03-31

**Authors:** Juan C. Alvarado, Verónica Fuentes-Santamaría, María C. Gabaldón-Ull, Tania Jareño-Flores, Josef M. Miller, José M. Juiz

**Affiliations:** ^1^Instituto de Investigación en Discapacidades NeurológicasAlbacete, Spain; ^2^Facultad de Medicina, Universidad de Castilla-La ManchaAlbacete, Spain; ^3^Grupo de Neurobiología de la Audición, Instituto de Investigaciones Biomédicas Alberto Sols, Consejo Superior de Investigaciones Científicas-Universidad Autónoma de MadridMadrid, Spain; ^4^Center for Hearing and Communication Research and Department of Clinical Neuroscience, Karolinska InstitutetStockholm, Sweden; ^5^Kresge Hearing Research Institute, University of MichiganAnn Arbor, MI, USA

**Keywords:** priming, conditioning, toughening, Wistar rats, cochlear nucleus, calcium

## Abstract

An appropriate conditioning noise exposure may reduce a subsequent noise-induced threshold shift. Although this “toughening” effect helps to protect the auditory system from a subsequent traumatic noise exposure, the mechanisms that regulate this protective process are not fully understood yet. Accordingly, the goal of the present study was to characterize physiological processes associated with “toughening” and to determine their relationship to metabolic changes in the cochlea and cochlear nucleus (CN). Auditory brainstem responses (ABR) were evaluated in Wistar rats before and after exposures to a sound conditioning protocol consisting of a broad-band white noise of 118 dB SPL for 1 h every 72 h, four times. After the last ABR evaluation, animals were perfused and their cochleae and brains removed and processed for the activity markers calretinin (CR) and neuronal nitric oxide synthase (nNOS). Toughening was demonstrated by a progressively faster recovery of the threshold shift, as well as wave amplitudes and latencies over time. Immunostaining revealed an increase in CR and nNOS levels in the spiral ganglion, spiral ligament, and CN in noise-conditioned rats. Overall, these results suggest that the protective mechanisms of the auditory toughening effect initiate in the cochlea and extend to the central auditory system. Such phenomenon might be in part related to an interplay between CR and nitric oxide signaling pathways, and involve an increased cytosolic calcium buffering capacity induced by the noise conditioning protocol.

## Introduction

It is well-known that noise overexposure induces hearing dysfunction and pathologies of the inner ear (Clark and Bohne, 1999; Daniel, 2007; Sliwinska-Kowalska and Davis, 2012; Basner et al., 2014). However, depending on the intensity and duration of the exposure, noise can result in two different outcomes. First, noise-induced hearing loss, the most common consequence of noise overexposure, is characterized by hair cells death along with a permanent increase in the auditory threshold that may be expressed as an immediate acoustic trauma or as a more progressive permanent noise-induced hearing loss (Clark and Bohne, 1999; Daniel, 2007; Le Prell et al., 2007; Basner et al., 2014). Second, non-damaging noise-induced “priming,” can result from stimulation with a low-level continuous noise (conditioning), or as a consequence of using higher-level interrupted noise exposure (“toughening”; Canlon et al., 1988; Subramaniam et al., 1991; Pukkila et al., 1997; Brown et al., 1998; Attanasio et al., 1999; Hamernik and Ahroon, 1999a; Kujawa and Liberman, 1999; Gourévitch et al., 2009). The conditioning and “toughening” phenomena have been observed in different experimental animal models and also in humans (Miyakita et al., 1992; Niu and Canlon, 2002a,b; Brashears et al., 2003). When experimental subjects are exposed to a conditioning/toughening noise stimulation protocol, the auditory threshold shift induced by the noise exposure is progressively reduced (Canlon et al., 1988; Subramaniam et al., 1991; Pukkila et al., 1997; Brown et al., 1998; Attanasio et al., 1999; Hamernik and Ahroon, 1999a; Kujawa and Liberman, 1999; Niu and Canlon, 2002a,b; Gourévitch et al., 2009).

This reduction in noise-induced threshold shift indicates that the noise-induced priming helps to protect the auditory system from a subsequent traumatic noise exposure by reducing the susceptibility to noise-induced hearing loss (Canlon et al., 1988; Subramaniam et al., 1991; Pukkila et al., 1997; Brown et al., 1998; Attanasio et al., 1999; Hamernik and Ahroon, 1999a; Gourévitch et al., 2009). However, the impact that conditioning and toughening have on regulating auditory pathway activity has not been investigated. Neither are the changes in the periphery induced by conditioning or toughening fully understood. It has been proposed that priming is not the consequence of systemic events but rather a local phenomenon initiated in the sound conditioned cochlea (Kujawa and Liberman, 1999; Yamasoba et al., 1999). In this regard, increases in the endogenous antioxidant system have been found in the lateral wall of the cochlea in chinchillas during noise conditioning (Jacono et al., 1998; Harris et al., 2006); and in the guinea pig conditioned cochlea, there is an upregulation of calmodulin levels in hair cells, associated with increases in intracellular calcium (Ca^2+^) levels (Zuo et al., 2008).

Noise-induced Ca^2+^ upregulation has also been observed in response to sound exposure conditions that produce either temporary (TTS) or permanent (PTS) auditory threshold shifts (Fridberger et al., 1998; Gröschel et al., 2011; Jacob et al., 2013). During noise overstimulation, increases in intracellular Ca^2+^ levels in hair cells and supporting cells occur concomitantly with increased stiffness and contraction of the Organ of Corti altering auditory function (Fridberger et al., 1998; Jacob et al., 2013). Noise-induced Ca^2+^ upregulation also occurs in the cochlear nucleus (CN) following TTS or PTS (Gröschel et al., 2011).

Ca^2+^, an activity-dependent ion, is involved in many essential neuronal functions in the central auditory system, including synaptic plasticity, neurotransmission, and cellular viability (Ghosh and Greenberg, 1995; Zirpel et al., 1998, 2000; Förster and Illing, 2000; Stack and Code, 2000; Zettel et al., 2001). In the cochlea, Ca^2+^ has been implicated in different processes including modification of the force generation by the hair bundle and the length of hair cells (Jacob et al., 2013). Additionally, it has also been involved in the regulation of blood flow and potassium homeostasis in the spiral ligament (Liang et al., 2003; Dai and Shi, 2011) and in the modulation of firing rate of spiral ganglion neurons (Lv et al., 2012; Davis and Crozier, 2015). Moreover, increases in metabolic activity in auditory neuronal pathways affected by noise overexposure may change Ca^2+^ flux and homeostasis and alter normal auditory function, leading to cell death if such alterations persist over time (Ghosh and Greenberg, 1995).

As noise-induced hearing loss and noise-induced priming are associated with increases in activity-dependent calcium signaling pathways, it could be expected that other signaling molecules such as nitric oxide (NO), which are related to Ca^2+^ concentration and cellular activity, may also play a significant role in noise-induced priming mechanisms. NO is an essential signaling molecule that in normal conditions plays a critical role in the maintenance of auditory function, participating in physiological processes that include neurotransmission, endolymph homeostasis, and blood flow regulation (Heinrich and Helling, 2012). However, depending on its expression levels, NO could be involved in cytotoxicity and even cell death, which may be relevant in hearing disorders (Heinrich and Helling, 2012). For example, noise-induced TTS in guinea pigs leads to upregulation of NO synthase (NOS) levels in the stria vascularis and in the spiral ligament (Chen et al., 2005; Heinrich and Helling, 2012). It is interesting to note that while auditory thresholds returned to normal values 2 days after overexposure, elevated NO levels persisted up to 5 days post-exposure (Chen et al., 2005; Heinrich and Helling, 2012). The fact that the constitutive isoforms, neuronal NOS (nNOS), and endothelial NOS (eNOS), are Ca^2+^/calmodulin dependent (Chen et al., 2005; Lin et al., 2011; Heinrich and Helling, 2012), suggest that increases in Ca^2+^ concentration that occur during noise-induced priming may induce parallel increases in NO levels within auditory nuclei thus, implicating NO in the mechanism of noise-induced priming.

Considering that activity dependent increases in Ca^2+^ influx could regulate the expression of molecules such as the calcium-binding protein calretinin (CR) and nNOS, it might be expected that changes in the levels of these molecules during the noise-induced toughening protocol are associated with modifications in auditory brainstem responses (ABR). To test this hypothesis, the aims of the present study were first to characterize the functional modifications in auditory thresholds, wave amplitudes, and latencies in Wistar rats after a noise-induced toughening paradigm by using ABR recordings. Second, to evaluate the expression of CR and nNOS in peripheral and central auditory structures in response to interrupted noise exposure. Third, to determine the temporal relationship between the functional and biochemical adaptations that could occur during toughening. To our knowledge, this is the first study evaluating the changes in CR and nNOS levels under specific conditions of noise-induced priming. Understanding the mechanisms underlying the toughening phenomenon may help to develop therapeutic strategies that, in turn, may reduce the impact of noise-induced hearing loss.

## Experimental procedures

### Animals

Six-month-old female Wistar rats (Charles River, Barcelona, Spain), were housed at the Universidad de Castilla–La Mancha animal facility (Albacete, Spain), under controlled conditions (temperature 22–23°C and humidity 60 ± 5%), on a 12 h light/dark cycle and food/water *ad libitum*. All procedures used were approved by the Ethics Committee on Animal Experimentation at the University of Castilla-La Mancha (Permit Number: PR-2013-02-03) and conformed to Spanish (R.D. 53/2013; Law 32/2007) and European Union (Directive 2010/63/EU) regulations for the care and use of animals in research.

### Auditory brainstem response (ABR) recordings

ABR recordings were performed, as described previously (Alvarado et al., 2012, 2014; Fuentes-Santamaría et al., 2012, 2013, 2014; Melgar–Rojas et al., 2015). Briefly, animals were placed into a sound-attenuating, electrically shielded booth (EYMASA/INCOTRON S.L., Barcelona, Spain) located inside a sound-attenuating room. Then, the rats were anesthetized with isoflurane (1 L/min O2 flow rate) at 4% for induction and 1.5–2% for maintenance. Subdermal needle electrodes (Rochester Electro–Medical, Tampa, FL, USA) were placed at the vertex (non–inverting) and in the right (inverting) and the left (ground) mastoids. Sound stimulation and recordings were performed using a BioSig System III (Tucker-Davis Technologies, Alachua, FL, USA). The stimuli, consisted of pure tone bursts sounds (5 ms rise/fall time without a plateau with a cos2 envelope delivered at 20/s) at seven different frequencies (0.5, 1, 2, 4, 8, 16, and 32 kHz). The sounds were generated digitally by the SigGenRP software (Tucker-Davis Technologies, Alachua, FL, USA) and the RX6 Piranha Multifunction Processor hardware (Tucker-Davis Technologies, Alachua, FL, USA), and were delivered into the external auditory meatus of the right ear using an EDC1 electrostatic speaker driver (Tucker–Davis Technologies) through an EC-1 electrostatic speaker (Tucker-Davis Technologies). Calibration was performed prior to the experiments using SigCal software (Tucker-Davis Technologies) and an ER-10B+ low noise microphone system (Etymotic Research Inc., Elk, Groove, IL, USA). Evoked responses were filtered (0.3–3.0 kHz), averaged (500 waveforms) and stored for offline analysis. During recordings, the temperature was monitored with a rectal probe and maintained at 37.5 ± 1°C using a non–electrical heating pad.

### Interrupted repetitive sound conditioning protocol

**Figure 1:**
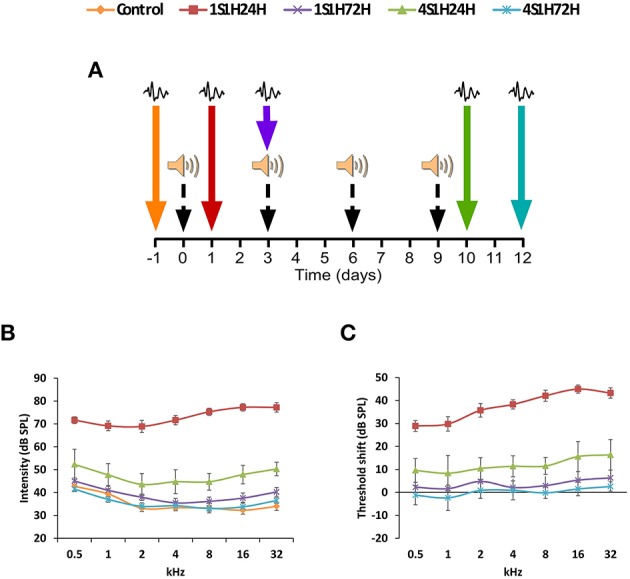
**(A)** Wistar rats were exposed to a noise-induced toughening protocol consisting of 1 h continuous white noise at 118 dB SPL in four different sessions, with a recovery time of 72 h between sessions. The ABR recordings were performed before the noise exposure and then at 24 (1S1H24H) and 72 h (1S1H72H) after the first session and at 24 (4S1H24H) and 72 h (4S1H72H) after the fourth session of the noise-induced toughening protocol. **(B)** Line graphs illustrating the relationship between the auditory thresholds and the frequencies evaluated in control and experimental groups. Note that the mean values in 1S1H24H rats were increased at all frequencies compared to control and the other experimental groups. Values in 4S1H24H animals were slightly increased mainly in the higher frequencies when compared to control and experimental rats. **(C)** In comparison to the control condition, the largest threshold shift was observed at 24 h after the first session and ranged from 28.93 to 44.96 dB. However, by 24 h after the fourth session the threshold shift varied from 8.35 to 16.34 dB. Color arrows indicate the time point at which the ABRs were performed and black dashed arrows show the corresponding sessions of the noise-induced toughening protocol.

The experimental protocol consisted of exposure to1 h continuous white noise at 118 dB SPL in four different sessions, with a recovery time of 72 h between sessions (Figure 1A). The sound was delivered inside a methacrylate reverberating chamber with 60 × 70 × 40 (length × width × height) cm with tilted and non-parallel walls in order to avoid standing waves and ensure a more homogeneous sound field. The chamber was placed into a double wall sound–attenuating booth located inside a sound–attenuating room. ABR recordings were performed in different animal groups before the first exposure (control, *n* = 4), at 24 h (1S1H24H, *n* = 4), and 72 h (1S1H72H, *n* = 4) after the first exposure and at 24 h (4S1H24H, *n* = 4) and 72 h (4S1H72H, *n* = 4) after the fourth noise overexposure. Animals of each group were sacrificed for histological assessment immediately following the ABR recording sessions.

### ABR data analysis

#### Thresholds

In order to evaluate the auditory threshold, the background activity was measured before the stimulus onset and the evoked responses were recorded in 5 dB steps descending from 80 dB sound pressure level (SPL). For each one of the frequencies evaluated, the auditory threshold was set as the stimulus intensity that evoked waveforms with a peak–to–peak voltage greater than two standard deviations (SD) of the background activity (Cediel et al., 2006; Garcia-Pino et al., 2010; Alvarado et al., 2012, 2014; Fuentes-Santamaría et al., 2012). In order to avoid acoustic trauma in control animals and any possible additional noise overstimulation in experimental rats during the ABR recordings, the maximum level of intensity was established at 80 dB (Gourévitch et al., 2009; Alvarado et al., 2012, 2014; Fuentes-Santamaría et al., 2013; Melgar–Rojas et al., 2015). Following the noise overstimulation protocol, if no evoked responses were obtained during the recording at 80 dB, the auditory thresholds were recorded at that value for statistical analysis (Subramaniam et al., 1992; Trowe et al., 2008; Alvarado et al., 2012, 2014; Fuentes-Santamaría et al., 2014; Melgar–Rojas et al., 2015).

#### Threshold shift

In each one of the frequencies tested, the threshold shift was determined as the differences between the auditory thresholds following the noise overstimulation protocol, minus the auditory thresholds in the control condition (Alvarado et al., 2012, 2014; Fuentes-Santamaría et al., 2012, 2013, 2014; Melgar–Rojas et al., 2015).

#### Wave amplitudes

They were defined as the peak-to-peak amplitude from the positive peak to the subsequent negative trough of each wave observed in the recording (Popelar et al., 2008; Church et al., 2010; Alvarado et al., 2012, 2014).

#### Contrast index

To facilitate comparisons among rats, the differences between wave amplitudes in the experimental condition and the control condition were normalized and quantified using a standard contrast index (Motter, 1994; Alvarado et al., 2007c, 2008, 2009b):

$$\begin{array}{l} \text{Contrast index}=\left(\text{WAEC}-\text{WACC}\right)∕\left(\text{WAEC}+\text{WACC}\right) \end{array}$$

Where WAEC is the wave amplitude in the experimental condition and WACC is the wave amplitude in the control condition. This index distributes values in the range from −1 to 1, with zero corresponding to an identical response in the experimental and control condition and positive and negative values corresponding to wave enhancement and wave depression following noise overstimulation, respectively.

#### Latencies

Two latencies were measured: (1) the positive latency comprising the time between the stimulus onset and the corresponding positive peak, and (2) the negative latency comprising the time between the stimulus onset and the negative trough (Chiappa et al., 1979; Chen and Chen, 1991; Gourévitch et al., 2009; Alvarado et al., 2012, 2014). Additionally, the positive and negative interpeak intervals between I-II, II-IV, and I-IV waves were calculated. The acoustic transit time of 0.5 ms between the speaker's diaphragm and the rat's tympanic membrane was included in the latencies.

### Immunohistochemistry

#### Specificity of antibodies

The antibodies used for detecting changes in neural and synaptic activity associated with sound stimulation are listed in Table 1. *The polyclonal anti-CR antibody* was raised against the human CR protein. It recognizes a single 31-kDa band that corresponds to the CR in western blots from the cochlear nucleus (Fuentes-Santamaría et al., 2005a). As this protein regulates calcium homeostasis by buffering the calcium that enters cells during synaptic activation (Baimbridge et al., 1992), disturbances in CR levels have been related to changes in afferent activity in auditory nuclei (Fuentes-Santamaría et al., 2003, 2005a; Alvarado et al., 2004, 2005, 2007a). *The polyclonal anti-neuronal NOS (nNOS) antibod*y recognizes a strong major band of 155 kDa on Western blot of rat cerebellum and cortex. Given the role of NO as a modulator of neurotransmitter release, this antibody has been used as a marker of neuronal excitability and firing (Esplugues, 2002; Fuentes-Santamaría et al., 2008). *The monoclonal anti-synaptophysin antibody* recognizes synaptophysin (38 kDa) in rat brain extracts using immunoblotting and immunohistochemistry. This protein is present in synaptic vesicles and has been associated with neurotransmitter release and used as a synaptic marker (Valtorta et al., 2004). The staining pattern of this antibody agrees with previous observations in the cochlear nucleus (Fuentes-Santamaría et al., 2005a, 2007a), superior olivary complex (Alvarado et al., 2004) and the inferior colliculus (Fuentes-Santamaría et al., 2003; Alvarado et al., 2007a).

**Table 1:** **List of primary antibodies**.

**Primary antibody**	**Immunogen**	**Host**	**Code/clone**	**Dilution**	**Manufacturer**
CR	Recombinant human CR	Rabbit	7699/3H	1:1500	Swant, Bellinzona, Switzerland
NOS	Amino acid residues 1414-1429 of the rat NOS-I protein	Rabbit	AB1552	1:1000	Millipore, Billerica, MA, USA
SYN	Rat retina	Mouse	clone SPV-38	1:200	Sigma Aldrich, MO, USA

#### Cochlea

At the corresponding survival time following noise exposure, control and experimental rats were anesthetized with an intraperitoneal injection of pentobarbital (200 mg/Kg). Following the loss of withdrawal reflexes, the animals were perfused transcardially with 0.9% saline wash followed by a fixative solution of 4% paraformadehyde in 0.1 M phosphate buffer (PB, pH 7.3). Cochleae were quickly removed from the temporal bone, post-fixed in 4% paraformaldehyde for 2 h and decalcified in 10% ethylenediamine tetraacetic acid (EDTA; pH 6.5) solution for 10 days. Then, they were cryoprotected in a solution of 30% sucrose overnight, embedded in a solution of 15% sucrose and 10% gelatin and frozen at 70°C by immersion in 2-propanol/dry ice bath. Cochleae were sectioned at 20 μm on a cryostat and mounted onto SuperFrost slides. After several rinses in phosphate-buffered saline (PBS) containing 0.2%Triton X-100 (Tx), cochlear sections were blocked for 1 h in PBS-Tx (0.2%) containing 10% normal goat serum (NHS) and incubated overnight at 4°C with primary antibodies for either CR, or nNOS or synaptophysin (SYN) diluted in a solution containing PBS-Tx (0.2%), pH 7.4. The following day, after four 15 min rinses in PBS-Tx (0.2%), sections were incubated for 2 h in either biotinylated goat anti-rabbit or biotinylated goat anti-mouse secondary antibodies (1:200, Vector Laboratories, Burlingame, CA, USA) and for 1 h in the avidin–biotin–peroxidase complex solution (ABC). Finally, sections were coverslipped using Cytoseal (Stephens Scientific).

#### Cochlear nucleus (CN)

Animals were anesthetized and perfused as previously indicated. The brains were removed from the cranium, and sucrose-cryoprotected for 48 h. Frozen sections 40 μm–thick were cut on a sliding microtome in the coronal plane. The immunohistochemistry procedure followed was the same as mentioned above for cochlear sections. Three sets of control experiments were performed to test the specificity of the immunohistochemistry detection system: (1) omission of the primary antibody by replacement with TBS-BSA; (2) omission of secondary antibodies; and (3) omission of ABC reagent. No immunostaining was detected under these conditions.

### Double immunofluorescence labeling

Two series of adjacent sections from these same animals were used for double-labeling studies. Both cochlear nucleus and cochlear sections were rinsed several times in 0.2% TBS-Tx and blocked for 1 h in the same buffer solution containing 10% normal goat serum. Then, cochlear sections were incubated overnight with either CR or nNOS primary antibodies while cochlear nucleus sections were incubated with either CR and SYN or nNOS and SYN primary antibodies. Following four 15 min rinses in 0.2% TBS-Tx, CN sections were incubated in a cocktail of fluorescently labeled secondary antibodies for 2 h at room temperature (1:200, anti-rabbit conjugated to Alexa 488 for CR and anti-mouse conjugated to Alexa 594 for SYN, or anti-rabbit conjugated to Alexa 488 for nNOS and anti-mouse conjugated to Alexa 594 for SYN; Molecular Probes, Eugene, OR, USA). After several rinses in TBS, sections were coverslipped with DAPI.

### Immunostaining data analysis

The immunostaining analysis was performed by using the public domain image analysis software Scion Image for Windows (version beta 4.0.2; developed by Scion Corp), as described elsewhere (Fuentes-Santamaría et al., 2003, 2005b, 2007a,b; Alvarado et al., 2004, 2009a). Immunostained cochlear and CN sections were examined via brightfield illumination using a Nikon Eclipse 80i photomicroscope (Nikon Instruments Europe B.V.). Images were captured using a DXM 1200C digital camera (Nikon Instruments Europe B.V.) that was attached to the microscope. The resultant color images of each field were digitized, and the resultant 8-bit red channel images, containing a grayscale of pixel intensities from 0 (white) to 255 (black), were used for the analysis. Assessors were blind as to whether cochlear or cochlear nucleus tissue was from unexposed or exposed animals.

#### Cochlear data analysis

The analysis of CR and nNOS immunostaining was performed on equally spaced mid-modiolar sections, 80 μm apart, for a total of 10 sections in each animal. For each section, two fields (dorsal and ventral) were sampled in each cochlear structure using a 60x objective. To perform an appropriate comparison of the immunostaining across samples, a macro was used to process and analyze the captured images (Alvarado et al., 2004). First, the images were normalized, then a threshold level was set at two SD above the mean gray level of the field, and immunostained profiles exceeding this threshold were identified as labeled.

#### CN data analysis

The CN subdivisions were defined based on previous studies (Cant and Benson, 2003). The analysis of the immunostaining was performed on equally spaced coronal sections, 160 μm apart, extending throughout the rostrocaudal dimension of the anterior (AVCN) and posterior (PVCN) ventral cochlear nucleus. A total of eight sections per side was analyzed in each animal. For each section, three fields (dorsal, middle, and ventral) were sampled using a 40x objective.

#### Immunostained indexes

The following indexes were measured: (1) the mean gray level of CR, nNOS, and SYN immunostaining, which was used as an indirect indicator of protein levels within cells or synaptic terminals. (2) The area of SYN immunostaining, which was calculated as the summed area of all profiles labeled above the threshold in each field, providing an estimate of the area of SYN.

#### Percentage of variation of the immunostaining

In order to determine whether there were statistically significant differences in the immunostaining for CR, nNOS, and SYN among control and experimental conditions, the percentage of variation of the immunostaining was calculated using an enhancement index, according to the following formula (Meredith and Stein, 1983; Alvarado et al., 2007b,c, 2009b):

$$\begin{array}{l} \text{\% of variation}=\left[\left(\text{IEC}-\text{ICC}\right)∕\left(\text{ICC}\right)\right]\times 100 \end{array}$$

Where IEC is the mean gray level or the immunostained area in the experimental condition following the noise protocol and ICC is the mean gray level or the immunostained area in the control condition before the noise overstimulation.

### Preparation of figures and statistical analysis

Photoshop (Adobe v5.5) and Canvas (Deneba v6.0) software packages were used to adjust the size, brightness and contrast of the images for the preparation of figures. All data are expressed as the means ± SEM. The measurements of the amplitudes and latencies were performed at 80 dB SPL unless otherwise indicated. Comparisons between groups were performed using a one-way analysis of variance (ANOVA) with Scheffé post-hoc analysis as necessary. Significance levels (α) and power (β) were set to 0.05 and 95%, respectively.

## Results

### Physiological findings

#### Auditory thresholds

The mean values of the auditory thresholds observed in the control condition (Figure 1B; Table 2) were similar to those described previously for Wistar rats (Jamesdaniel et al., 2009; Church et al., 2010; Alvarado et al., 2012, 2014; Pilati et al., 2012; Melgar–Rojas et al., 2015). By 24 h after the first session of the sound conditioning protocol (1S1H24H), there was an increase of the auditory thresholds at all frequencies evaluated when compared to the control condition (Figure 1B; Table 2). The auditory thresholds retuned to the control value at 72 h after the first session (1S1H72H) of the sound conditioning protocol (Figure 1B; Table 2). In contrast to that observed at 24 h after the first session, at 24 h after the fourth session (4S1H24H) there was only a slight increase in auditory thresholds evident at medium and high frequencies (Figure 1B; Table 2). Similarly, by 72 h after the fourth session of the sound conditioning protocol (4S1H72H) the auditory thresholds were similar to those found in control rats (Figure 1B; Table 2). After the first session of sound conditioning, the threshold shifts at 24 h (1S1H24H) and at 72 h (1S1H72H) ranged from 28.93 to 44.96 dB and from 1.63 to 6.30 dB, respectively (Figure 1C). Twenty four hours after the fourth session of the sound conditioning protocol (4S1H24H), the threshold shift varied from 8.35 to 16.34 dB, decreasing at 72 h (4S1H72H) from −2.37 to 2.55 (Figure 1C). The analysis of variance demonstrated a significant interaction between the number of exposures (1 vs. 4) and the time post-exposure and the auditory thresholds (Table 2). Accordingly, the highest auditory thresholds were found at the earliest noise exposure at the earliest time (1S1H24H; Figures 1B,C), reflecting the progressive resistance or toughening effect that occurred under the sound conditioning protocol.

**Table 2:** **ANOVA analysis of the interaction between the time post-exposure and the auditory thresholds**.

	**0.5**	**1**	**2**	**4**	**8**	**16**	**32**
**FREQUENCIES (kHz)**							
Control (1)	42.74 ± 1.99	39.37 ± 2.20	33.16 ± 1.39	33.37 ± 1.33	33.21 ± 2.11	32.26 ± 1.69	33.95 ± 1.83
1S1H24H (2)	71.67 ± 1.46	69.17 ± 2.15	68.89 ± 2.64	71.67 ± 2.02	75.28 ± 1.54	77.22 ± 1.60	77.01 ± 2.07
1S1H72H (3)	45.00 ± 1.38	41.00 ± 1.99	37.92 ± 1.44	35.50 ± 1.92	36.17 ± 1.86	37.58 ± 2.21	40.25 ± 1.95
4S1H24H (4)	52.29 ± 6.67	47.71 ± 4.96	43.57 ± 4.72	44.71 ± 5.21	44.62 ± 3.70	47.86 ± 4.06	50.29 ± 2.94
4S1H72H (5)	41.50 ± 1.04	37.05 ± 1.22	34.10 ± 1.47	34.25 ± 1.75	33.03 ± 1.08	33.75 ± 1.97	36.50 ± 1.26
ANOVA	*F*_(4, 57)_ = 27.07 (^***^)	*F*_(4, 57)_ = 23.37 (^***^)	*F*_(4, 57)_ = 38.44 (^***^)	*F*_(4, 57)_ = 39.03 (^***^)	*F*_(4, 57)_ = 51.92 (^***^)	*F*_(4, 57)_ = 54.48 (^***^)	*F*_(4, 57)_ = 51.59 (^***^)
**SIGNIFICANCE LEVELS**							
1 vs. 2	^***^	^***^	^***^	^***^	^***^	^***^	^***^
1 vs. 3	NS	NS	NS	NS	NS	NS	NS
1 vs. 4	NS	NS	NS	^**^	^**^	^***^	^***^
1 vs. 5	NS	NS	NS	NS	NS	NS	NS
2 vs. 3	^***^	^***^	^***^	^***^	^***^	^***^	^***^
2 vs. 4	^***^	^***^	^***^	^***^	^***^	^***^	^***^
2 vs. 5	^***^	^***^	^***^	^***^	^***^	^***^	^***^
3 vs. 4	NS	NS	NS	^**^	^**^	^***^	^***^
3 vs. 5	NS	NS	NS	NS	NS	NS	NS
4 vs. 5	NS	NS	NS	^**^	^**^	^***^	^***^

** *p < 0.01*;

*** *p < 0.001*;

*NS, No significance*.

#### Waves amplitudes

**Figure 2:**
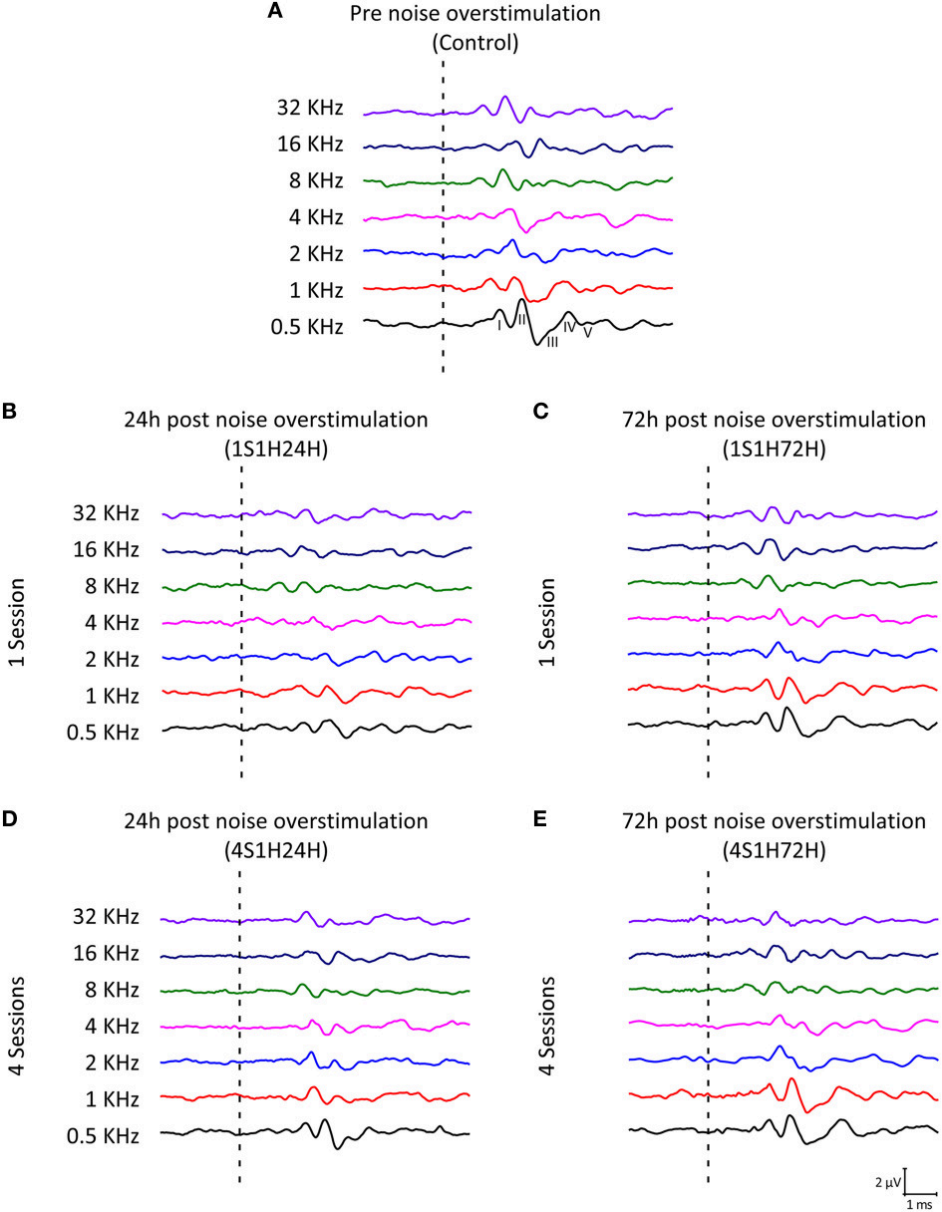
**Line graphs showing examples of ABR recordings in control and experimental rats at 80 dB SPL for all frequencies evaluated. (A)** In the control group, the recordings displayed a distinctive pattern characterized by 4–5 evoked waveforms after the stimulus onset. **(B)** By 24 h after the first session of the noise-induced toughening protocol there was a significant decrease in the amplitude of all waveforms in the ABR. **(C)** The amplitudes apparently returned to values close to the normal condition in 1S1H72H animals. **(D)** In 4S1H24H rats, although there was a slightly decrease in the waves amplitude, it was not as evident as in 1S1H24H rats. **(E)** The recordings in the 4S1H72H condition were similar to those observed in control. Dashed lines indicate the stimulus onset. Stimulus intensity = 80 dB SPL.

**Figure 3:**
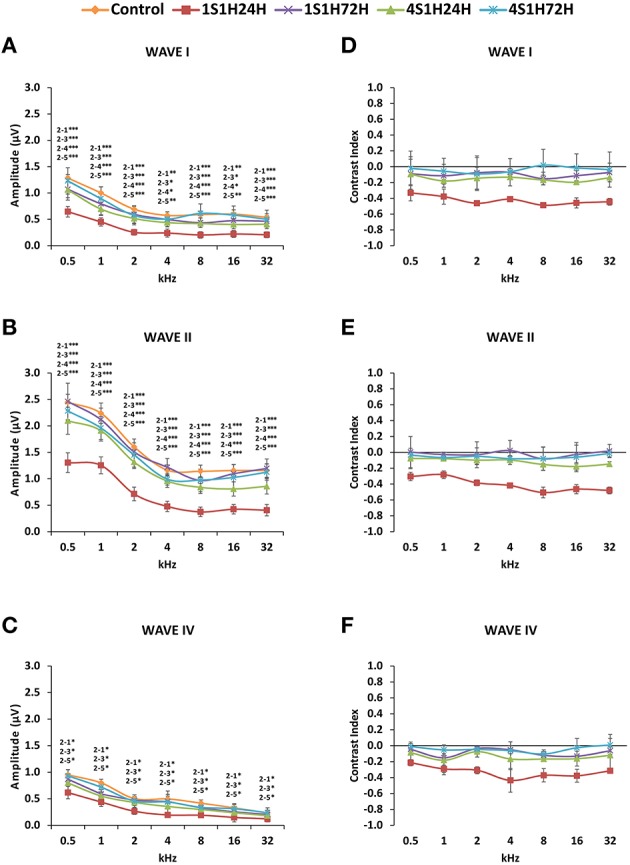
**Line graphs showing the amplitudes (in μV) and contrast index of waves I, II, and IV plotted as a function of frequencies in control and experimental animals**. Before sound stimulation, wave amplitudes were larger in the lower frequencies and smaller at medium and higher frequencies, being wave II the largest of all **(A–C)**. However, by 24 h after the first session there was a significant decrease in all waves amplitudes **(A–C)**, with contrast indexes below −0.2 in all waves and at all frequencies **(D–F)**. The amplitudes **(A–C)** as well as the contrast index **(D–F)** were recovered by the fourth session. Stimulus intensity = 80 dB SPL. (1) Control; (2) 1S1H24H; (3) 1S1H72H; (4) 4S1H24H; (5) 4S1H72H. ^*^p < 0.05; ^**^p < 0.01; ^***^p < 0.001.

As it is shown in Figure 2, the ABR recordings in control and experimental rats showed the four to five characteristic evoked waves described previously in Wistar rats (Overbeck and Church, 1992; Church et al., 2010, 2012a,b; Alvarado et al., 2012, 2014). The wave II was the largest, followed by waves I, IV, and V, while the wave III was the smallest (Figure 2A). Despite of the similarities among groups, a decrease in all waves at all frequencies was observed following each of the exposures at each of the measurement times, most obvious at 24 h after the first noise conditioning exposure (1S1H24H; Figure 2B) when compared to controls (Figure 2A). Comparison of the mean values of the amplitudes of the largest waves of the ABR (I, II, and IV), revealed that the amplitudes in the 1S1H24H rats were lower than those observed in control and the other exposure groups (Figures 3A–C) for all waves and frequencies. The contrast index demonstrated depression in the amplitudes of the evoked responses by 24 h after the first session of sound conditioning (1S1H24H) relative to to the control condition in waves I, II, and IV, although it was more evident at higher frequencies and in the waves I and II (Figures 3D–F). Similarly to what was observed for the auditory thresholds, ANOVA analysis indicated a statistically significant interaction between time post-exposure and the amplitude of the ABR waveform. Specifically, for the wave I (Table 3, Figure 3A) and wave II (Table 3, Figure 3B), the amplitudes along all frequencies in 1S1H24H rats were significant lower than those found in the control group and in other experimental rats (1S1H72H, 4S1H24H, and 4S1H72H). For the wave IV (Table 3, Figure 3C), the amplitudes in the 1S1H24H group were significantly lower than the amplitudes in control, 1S1H72H and 4S1H72H groups, but not different from those seen at 24 h after the fourth session (4S1H24H) of the sound conditioning protocol (Table 3, Figure 3C).

**Table 3:** **ANOVA analysis of the interaction between the time post-exposure and the wave amplitudes**.

**Waves**	**0.5**		**1**		**2**		**4**		**8**		**16**		**32**	
**FREQUENCIES (kHz)**														
I	*F*_(4, 57)_ = 6.67	^***^	6.82	^***^	7.57	^***^	4.67	^**^	8.75	^***^	4.85	^**^	6.37	^***^
II	*F*_(4, 57)_ = 6.32	^***^	5.47	^***^	7.26	^***^	7.42	^***^	7.15	^***^	7.32	^***^	6.99	^***^
IV	*F*_(4, 57)_ = 3.47	^*^	3.62	^*^	3.51	^*^	6.32	^**^	5.94	^**^	3.72	^*^	2.86	^*^

* *p < 0.05*;

** *p < 0.01*;

*** *p < 0.001*.

#### Wave latencies

**Figure 4:**
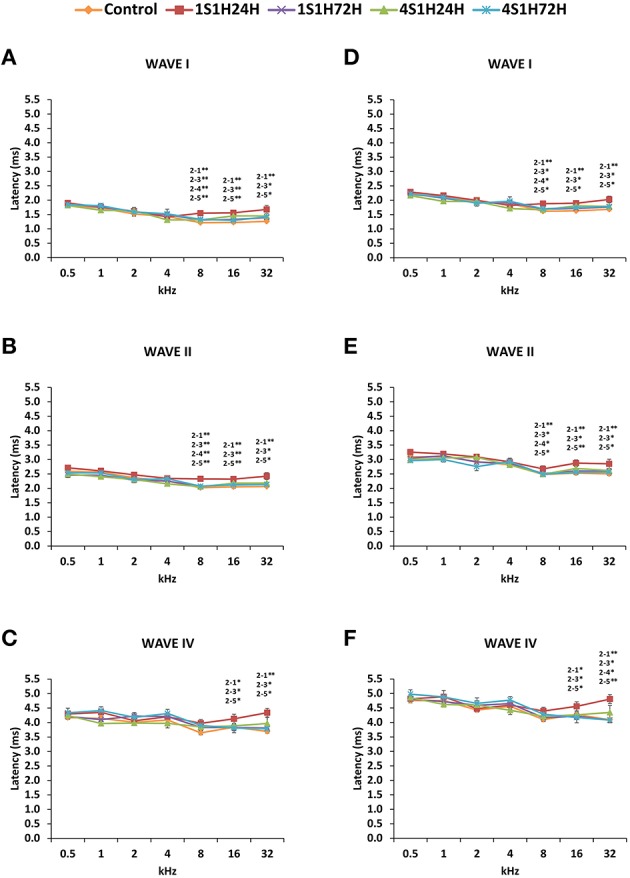
**Line graphs illustrating the latencies of the positive and negative peaks (ms) of waves I, II, and IV plotted as a function of the frequencies in control and experimental conditions**. In 1S1H24H rats, there were significantly longer positive and negative peak latencies at the middle and high frequencies **(A–F)**. These values returned to normal at the fourth session of noise exposure **(A–F)**. As indicated in the Methods Section, 0.5 ms of acoustic transit time between the speaker's diaphragm and the rat's tympanic membrane was included in the latencies. Stimulus intensity = 80 dB SPL. (1) Control; (2) 1S1H24H; (3) 1S1H72H; (4) 4S1H24H; (5) 4S1H72H. ^*^p < 0.05; ^**^p < 0.01.

**Figure 5:**
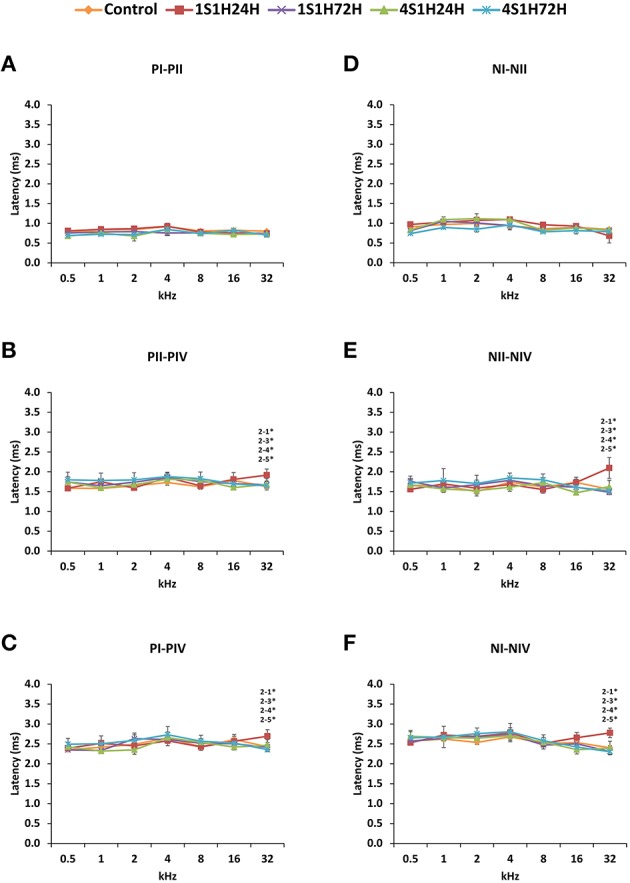
**Line graphs showing the positives and negatives interpeak intervals (ms) of waves I, II, and IV plotted as a function of the frequencies in control and experimental animals**. Whereas no differences were found between waves I and II **(A,D)**, significantly longer positives and negatives interpeak intervals at 32 kHz were observed between waves II and IV **(B,E)** and between waves I and IV **(C,F)** by 24 h after the first session. These differences were no longer present by the fourth session of noise overstimulation. Stimulus intensity = 80 dB SPL. (1) Control; (2) 1S1H24H; (3) 1S1H72H; (4) 4S1H24H; (5) 4S1H72H. ^*^p < 0.05.

The latencies of the positive and negative peaks (Figure 4) as well as the interpeak intervals (Figure 5) observed in the control condition were similar to those described elsewhere for Wistar rats (Overbeck and Church, 1992; Church et al., 2010, 2012a,b; Alvarado et al., 2012, 2014). Following the sound conditioning protocol there was a significant effect between time post-exposure and wave latencies. For wave I there were significant differences at 8, 16, and 32 kHz for both the positive (Figure 4A, Table 4) and the negative (Figure 4D, Table 4) peak latencies. At 8 kHz, the latencies of both positive and negative peaks by 24 h after the first session of sound conditioning (1S1H24H) were longer than the latencies measured in the control and the other experimental groups (Figures 4A,D, Table 4). However, at 16 and 32 kHz the latencies of the positive and negative peaks in 1S1H24H rats were longer than in control, 1S1H72H and 4S1H72H, but not significantly different from latencies recorded in 4S1H24H (Figures 4A,D, Table 4) rats. Similarly, for wave II, in the 1S1H24H group statistically significant, longer positive (Figure 4B, Table 4) and negative (Figure 4E, Table 4) peak latencies were found at 8 kHz when compared to control and the other experimental groups, and at 16 and 32 kHz compared to control, 1S1H72H and 4S1H72H (Figures 4B,E, Table 4) animals. For wave IV, there were also statistically significant longer positive peak latencies in the 1S1H24H condition compared to control, 1S1H72H and 4S1H72H groups at 16 and 32 (Figure 4C, Table 4) and at 16 kHz in the negative peak latencies (Figure 4F, Table 4). Whereas, at 32 kHz the negative peak latencies for wave IV were significantly longer when the 1S1H24H condition was compared to the control or the other experimental groups (Figure 4F, Table 4).

**Table 4:** **ANOVA analysis of the interaction between the time post-exposure and the wave latencies**.

**Waves**		**Frequencies (kHz)**												
		**0.5**	**1**		**2**		**4**		**8**		**16**		**32**	
**POSITIVE WAVE LATENCIES**														
I	*F*_(4, 57)_ = 1.31	NS	0.49	NS	0.35	NS	2.04	NS	5.23	^**^	3.78	^**^	4.14	^**^
II	*F*_(4, 57)_ = 2.60	NS	1.69	NS	1.15	NS	1.47	NS	5.06	^**^	3.20	^*^	3.65	^*^
IV	*F*_(4, 57)_ = 0.94	NS	2.16	NS	1.88	NS	0.77	NS	1.60	NS	3.89	^*^	5.12	^**^
**NEGATIVE WAVE LATENCIES**														
I	*F*_(4, 57)_ = 1.83	NS	1.20	NS	0.25	NS	1.37	NS	3.67	^*^	2.99	^*^	3.06	^*^
II	*F*_(4, 57)_ = 1.81	NS	1.18	NS	1.61	NS	0.52	NS	5.05	^**^	3.11	^*^	3.21	^*^
IV	*F*_(4, 57)_ = 0.68	NS	1.39	NS	0.96	NS	0.56	NS	1.16	NS	3.98	^*^	6.98	^***^
**INTERPEAK LATENCIES**														
PI-PII	*F*_(4, 57)_ = 2.18	NS	1.14	NS	2.08	NS	1.52	NS	1.38	NS	2.03	NS	1.56	NS
NI-NII	*F*_(4, 57)_ = 2.54	NS	1.47	NS	1.01	NS	1.57	NS	2.16	NS	0.58	NS	0.33	NS
PII-PIV	*F*_(4, 57)_ = 0.63	NS	1.80	NS	1.31	NS	0.41	NS	0.44	NS	0.72	NS	4.12	^*^
NII-NIV	*F*_(4, 57)_ = 1.10	NS	0.58	NS	1.48	NS	0.16	NS	0.16	NS	1.17	NS	4.27	^**^
PI-PIV	*F*_(4, 57)_ = 1.57	NS	1.95	NS	1.47	NS	0.49	NS	1.04	NS	0.41	NS	3.43	^**^
NI-NIV	*F*_(4, 57)_ = 1.31	NS	1.01	NS	1.38	NS	0.52	NS	0.78	NS	0.87	NS	3.60	^*^

* *p < 0.05*;

** *p < 0.01*;

*** *p < 0.001*;

*NS, No significance*.

Regarding the evaluation of the interpeak intervals (Figures 5A,D, Table 4), the ANOVA test revealed a significant interaction between post-exposure time and the positive and negative interpeak intervals of waves II-IV and I-IV at 32 kHz (Figures 5B–F, Table 4). Specifically, at 24 h after the first session of the sound conditioning protocol (1S1H24H) both the positive and negative interpeak intervals were longer at 32 kHz between waves II-IV (Figures 5B,E, Table 4) and between waves I-IV (Figures 5C,F, Table 4) in control and experimental rats.

### Histological findings

#### CR immunostaining in the cochlea

**Figure 6:**
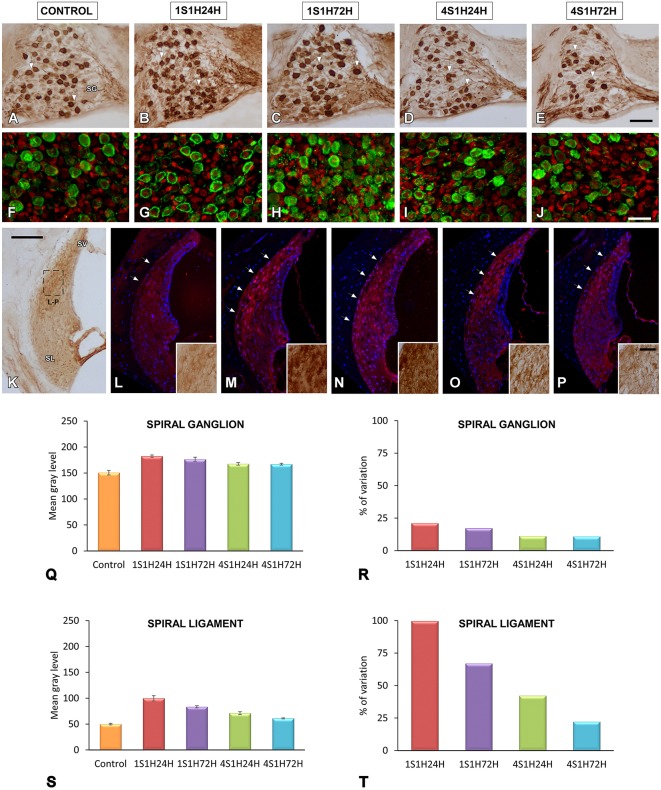
**Digitized images illustrating CR immunoreactivity in the spiral ganglion (A–J) and the spiral ligament (K–P) in control and experimental Wistar rats**. Before noise overstimulation, CR immunostaining in the spiral ganglion was characterized by dark immunopositive somata and fibers that were lightly stained throughout the ganglion **(A,F)**. Meanwhile, in the spiral ligament, the staining was present in type I fibrocytes that were moderately stained and distributed in the dorsolateral area (arrows and inset in **L**). Following the noise-induced toughening protocol, there was an increase in the immunostaining in spiral ganglion neurons and neuropil in 1S1H24H **(B,G)** rats that was still present at 1S1H72H **(C,H)**, 4S1H24H **(D,I)**, and 4S1H72H **(E,J)** animals relative to the control condition. Similar increases were also observed in the spiral ligament (arrows and inset in **M–P**) when compared to the control condition (arrows and inset in **K,L**). Bar graphs indicate the mean gray levels of CR immunostaining and its corresponding percentage of variation in the spiral ganglion **(Q,R)** and spiral ligament **(S,T)** in experimental and control rats. The square box in **(K)** indicates the approximate location of the high-magnification images illustrated in **(L–P)**. Scale bars represent 100 μm in **(K)**, 50 μm in **(E)**, and 25 μm in **(J,P)**. SG, spiral ganglion; SL, spiral ligament; SV, stria vascularis.

The activity-dependent calcium binding protein, CR, an excellent marker of cells with high rates of activity such as auditory neurons (Zirpel et al., 1998, 2000; Förster and Illing, 2000; Stack and Code, 2000; Zettel et al., 2001), was evaluated in the spiral ganglion and the spiral ligament. Qualitatively, under control conditions, spiral ganglion CR immunostaining was characterized by moderately stained somata and fibers throughout the whole ganglion (Figures 6A,F). Although the quantification of the proportion of labeled/unlabeled CR cells was not performed in the present study, we estimate that about half of these ganglion neurons (~50%) were CR immunostained while the other half were unstained cells or cells expressing low CR levels (Figures 6A,F).

At 24 h following the first session of the sound conditioning protocol (1S1H24H) there was an increase in the number of the immunopositive cells and fibers as well as CR intensity levels compared to the control rats (Figures 6B,G). Such an increase in the CR immunostaining was still present in the 1S1H72H condition (Figures 6C,H). It decreased in 4S1H24H (Figures 6D,I) and 4S1H72H (Figures 6E,J) animals compared to 1S1H24H and 1S1H72H groups, but remained elevated compared to the control condition (Figures 6A,F).

To quantify these observations, an analysis of the mean gray level of CR immunostaining was performed in the spiral ganglion. An ANOVA test showed significant interaction between the post-exposure time and the mean gray level of the immunostaining (Table 5). Accordingly, there was a statistically significant increase in CR immunostaining in the spiral ganglion in 1S1H24H, 1S1H72H, 4S1H24H, and 4S1H72H groups when compared to control animals (Figure 6Q, Table 5). No significant differences were found among any of the experimental groups after noise exposure (Figure 6Q, Table 5). The percentage of variation of CR immunostaining in experimental animals relative to control rats decreased progressively from 21.14% to 10.91% (Figure 6R).

**Table 5:** **Mean gray level of CR immunostaining in the spiral ganglion and spiral ligament in control and experimental animals**.

		**Spiral ganglion**	**Spiral ligament**
	Control (1)	150.67 ± 4.35	49.98 ± 1.57
	1S1H24H (2)	182.53 ± 2.23	99.81 ± 5.18
	1S1H72H (3)	176.59 ± 3.98	83.51 ± 2.10
	4S1H24H (4)	167.64 ± 2.46	71.07 ± 2.87
	4S1H72H (5)	167.11 ± 1.76	61.07 ± 1.02
	ANOVA	*F*_(4, 27)_ = 15.45 ^***^	*F*_(4, 25)_ = 45.02 ^***^
		**Significance levels**	
Statistical Comparison	1 vs. 2	^***^	^***^
	1 vs. 3	^***^	^***^
	1 vs. 4	^*^	^**^
	1 vs. 5	^*^	^*^
	2 vs. 3	NS	^**^
	2 vs. 4	NS	^***^
	2 vs. 5	NS	^***^
	3 vs. 4	NS	NS
	3 vs. 5	NS	^***^
	4 vs. 5	NS	NS

*Values are means ± standard errors*.

* *p < 0.05*;

** *p < 0.01*;

*** *p < 0.001*;

*NS, No significant*.

In the spiral ligament, CR immunostaining in the control animals was present in type I fibrocytes which were moderately stained and localized mostly in the dorsolateral region (Figure 6K; arrows and inset in Figure 6L). Similar to what occurs in the spiral ganglion, CR immunostaining peaked in 1S1H24H animals (arrows and inset in Figure 6M) and decreased in 1S1H72H (arrows and inset in Figure 6N), 4S1H24H (arrows and inset in Figure 6O), and 4S1H72H (arrows and inset in Figure 6P) rats when compared to the 1S1H24H condition. Note however, that the immunostaining in 4S1H72H animals was still darker than in the control condition (compare Figure 6P with Figure 6L). The ANOVA test of the mean gray level data also revealed a significant effect of the time post-exposure on the mean gray level of CR immunostaining in the spiral ligament (Table 5). The conditioning exposures resulted in a significant increase in the mean gray level for all measurement times compared to the control condition. The increased CR immunostaining in 1S1H24H animals was significantly higher than in the other experimental groups (Figure 6S, Table 5). The percentage of increase in CR immunostaining decreased progressively from 99.68% for the 1S1H24H condition to 22.19% for the 4S1H72H condition compared to the control group (Figure 6T).

#### nNOS immunostaining in the cochlea

**Figure 7:**
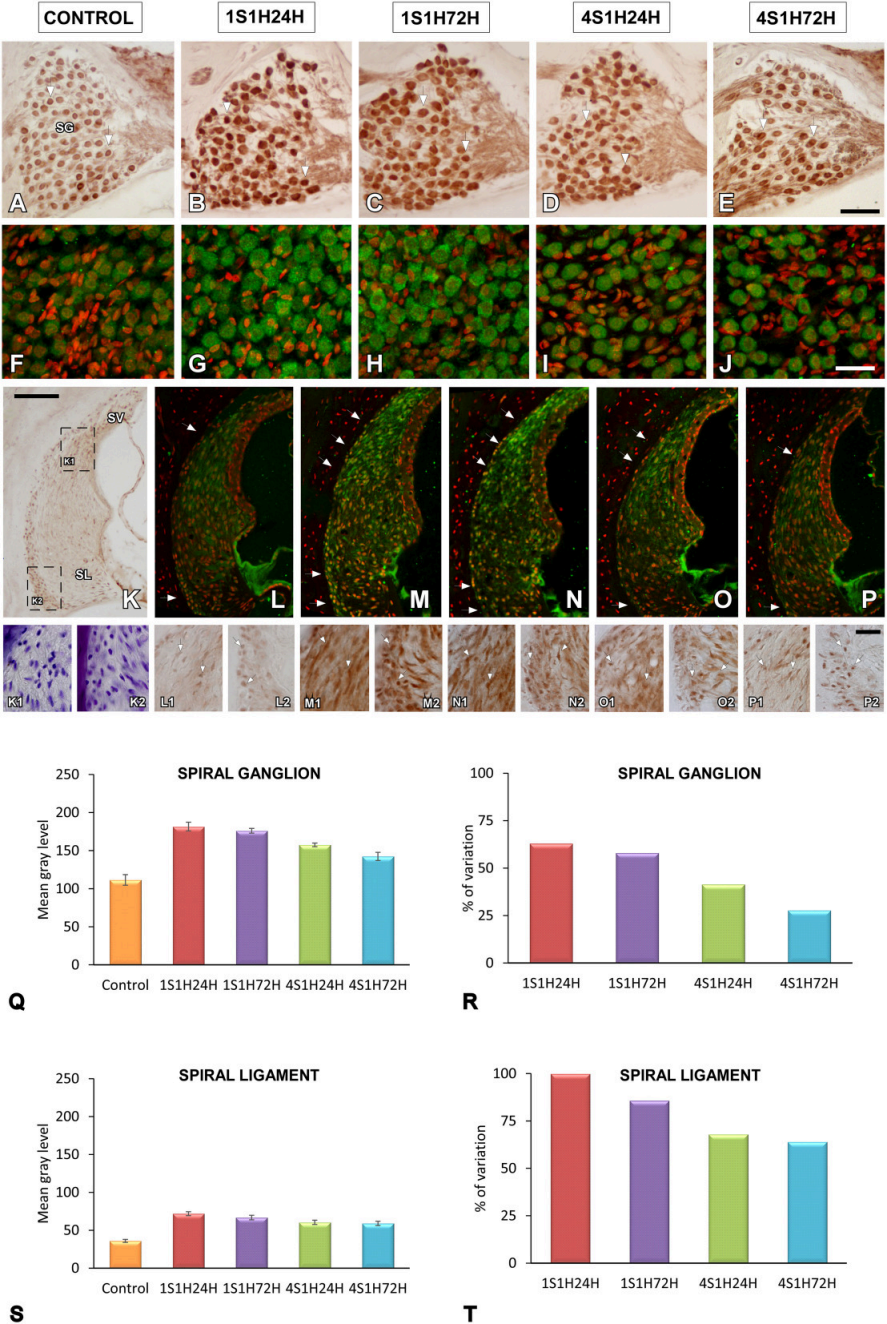
**Digitized images illustrating nNOS immunostaining in the spiral ganglion (A–J) and the spiral ligament (K–P) in control and experimental Wistar rats**. Before sound conditioning, nNOS immunostaining in spiral ganglion was located within neurons that were lightly immunostained **(A,F)**, whereas in the spiral ligament, the staining was weak and mainly distributed in the region where types I and III fibrocytes were found (arrows in **L**; also see **L1-L2)**. In the 1S1H24H condition, nNOS levels in the spiral ganglion were upregulated **(B,G)** and persisted elevated in 1S1H72H **(C,H)**, 4S1H24H **(D,I)** and 4S1H72H **(E,J)** groups when compared to control rats. A similar upregulation was also observed in the spiral ligament (arrows in **K–P** and arrows in **K1–P2**). Bar graphs indicate the mean gray levels of nNOS immunostaining in the spiral ganglion **(Q)** and the spiral ligament **(S)** and their corresponding percentage of variation **(R,T)**. The squares boxes in **(K)** indicate the approximate location of the high-magnification images illustrated in **(L–P)**. Scale bars represent 100 μm in **(K)**, 50 μm in **(E)**, and 25 μm in **(J)** and **(P2)**. SG, spiral ganglion; SL, spiral ligament; SV, stria vascularis.

An upregulation in CR levels during the sound conditioning protocol indicates an increase in intracellular calcium concentration associated with increased activity in the auditory system (Ghosh and Greenberg, 1995; Zirpel et al., 1998, 2000; Förster and Illing, 2000; Stack and Code, 2000; Zettel et al., 2001). Considering the role of NOS as a signaling molecule related to both calcium concentration and neuronal activity, its expression levels were evaluated following exposure to the conditioning stimuli. In the spiral ganglion, nNOS immunostaining followed a similar distribution pattern as described for CR. Before noise stimulation, nNOS-stained neurons were lightly immunostained (Figures 7A,F) while after the sound conditioning protocol, nNOS levels within neurons were increased in the 1S1H24H (Figures 7B,G) group and remained elevated in 1S1H72H (Figures 7C,H), 4S1H24H (Figures 7D,I), and 4S1H72H (Figures 7E,J) conditions compared to control animals. ANOVA demonstrated a significant interaction between the time post-exposure and the mean gray level of nNOS immunostaining in the spiral ganglion (Table 6). The post-hoc test revealed that in all the experimental conditions the increase in the mean gray level of nNOS immunostaining was statistically significant, higher than the values in control (Figure 7Q, Table 6) animals. Additionally, the mean gray level in the 4S1H72H group, although higher than in control rats, was significantly lower than in the other experimental conditions (Figure 7Q, Table 6). The percentage of variation of the upregulation in nNOS immunostaining decreased from 62.89% for 1S1H24H rats to 27.71% for 4S1H72H rats (Figure 7R).

**Table 6:** **Mean gray level of NOS immunostaining in the spiral ganglion and Spiral ligament in control and experimental animals**.

		**Spiral ganglion**	**Spiral ligament**
	Control (1)	111.51 ± 6.91	35.96 ± 2.03
	1S1H24H (2)	181.63 ± 5.75	71.90 ± 2.58
	1S1H72H (3)	176.16 ± 3.09	66.78 ± 3.04
	4S1H24H (4)	157.61 ± 2.39	60.39 ± 2.81
	4S1H72H (5)	142.41 ± 5.46	58.97 ± 2.81
	ANOVA	*F*_(4, 20)_ = 28.85 ^***^	*F*_(4, 20)_ = 26.51 ^***^
		**Significance levels**	
Statistical comparison	1 vs. 2	^***^	^***^
	1 vs. 3	^***^	^***^
	1 vs. 4	^***^	^***^
	1 vs. 5	^**^	^***^
	2 vs. 3	NS	NS
	2 vs. 4	NS	NS
	2 vs. 5	^**^	^*^
	3 vs. 4	NS	NS
	3 vs. 5	^*^	NS
	4 vs. 5	NS	NS

*Values are means ± standard errors*.

* *p < 0.05*;

** *p < 0.01*;

*** *p < 0.001*;

*NS, No significant*.

The changes in nNOS immunostaining in the spiral ligament after the sound conditioning protocol were similar to those observed in the spiral ganglion. In unexposed rats, the staining was weak and distributed in the dorsolateral part of the ligament which corresponds to the region where types I and III fibrocytes are located (Figures 7K,K1,K2; also see arrows in Figures 7L,L1,L2). In all the experimental groups, nNOS-immunostaining within cells was upregulated when compared to control rats (arrows in Figures 7K–P,K1–P2). Confirming these observations, the statistical evaluation of the immunostaining indicated a significant interaction between the time post-exposure and the mean gray level of the nNOS immunostaining in the spiral ligament (Table 6). All the experimental groups showed mean gray levels significantly higher than the values of control rats (Figure 7S, Table 6). The comparison among experimental animals also indicated that the mean gray level in the 1S1H24H condition was significantly higher than that in 4S1H72H (Figure 7S, Table 6) rats. The percentage of variation of the staining relative to the control condition ranged from 99.96 to 63.99% for the 1S1H24H and 4S1H72H groups (Figure 7T).

#### CR immunostaining in the AVCN and PVCN

**Figure 8:**
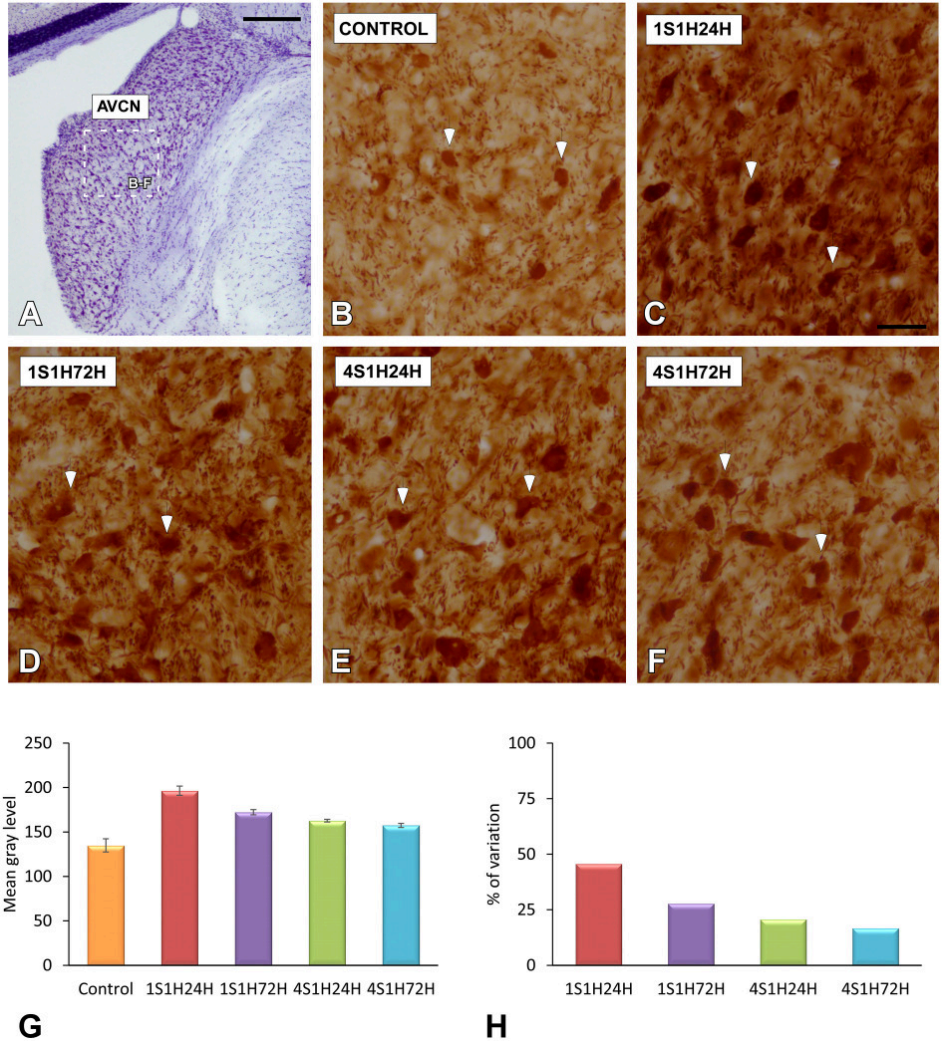
**Digitized images illustrating CR immunostaining in the AVCN in control and experimental Wistar rats**. At 24 h after the first session of sound conditioning, CR levels in both neurons and neuropil were increased (arrows in **C**), and continued elevated in 1S1H72H (arrows in **D**), 4S1H24H (arrows in **E**), and 4S1H72H (arrows in **F**) conditions when compared to control rats (arrows in **B**). Bar graphs show the mean gray level of CR immunostaining **(G)** and its percentage of variation **(H)** in the experimental conditions relative to control. The square box in **(A)** indicates the approximate location of the high-magnification images shown in **(B–F)**. Scale bars represent 250 μm in **(A)**, and 50 μm in **(C)**.

**Figure 9:**
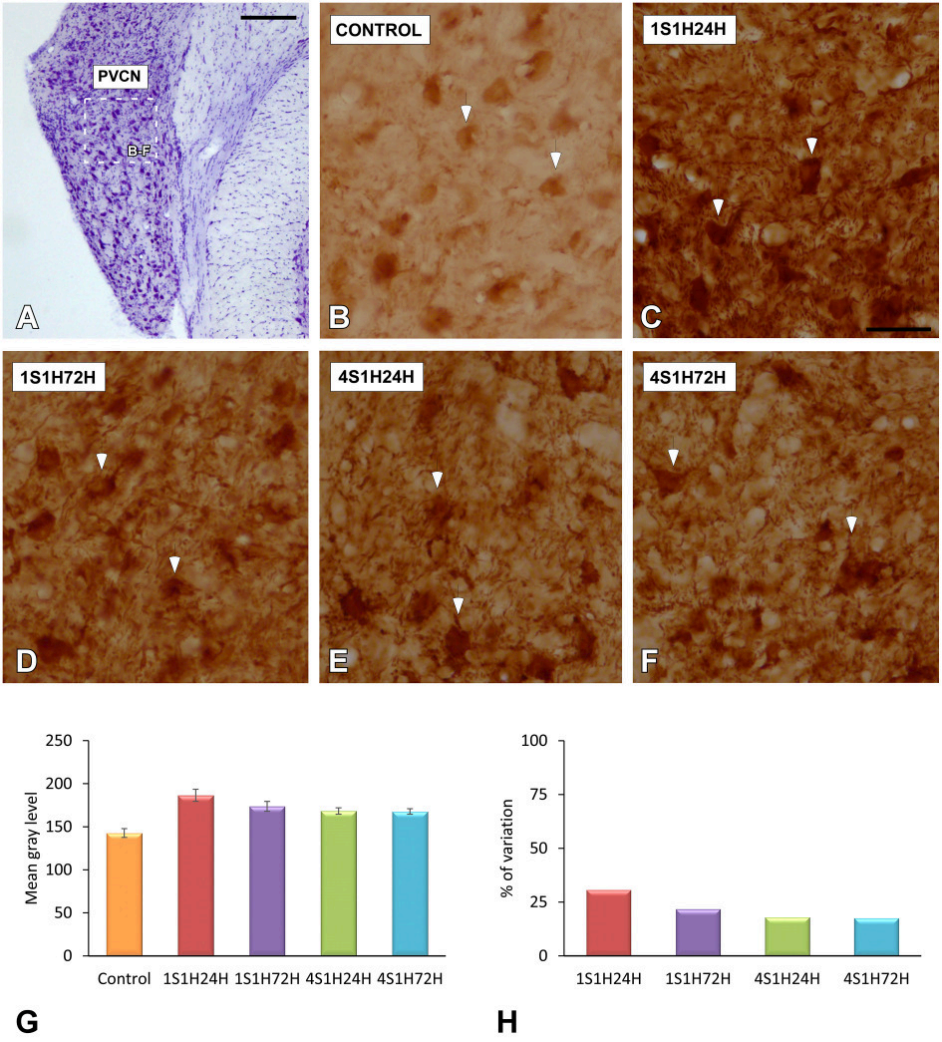
**Digitized images illustrating CR immunostaining in the PVCN in control and experimental Wistar rats**. Similar to that in the AVCN, CR levels in both neurons and neuropil were augmented in all the experimental conditions (arrows in **C–F**) when compared to control rats (arrows in **B**). Bar graphs show the mean gray level of CR immunostaining **(G)** and the percentage of variation **(H)** in the experimental conditions with respect to control. The square box in **(A)** indicates the approximate location of the high-magnification images shown in **(B–F)**. Scale bars represent 250 μm in **(A)**, and 50 μm in **(C)**.

As upregulation in CR levels in the cochlea following the sound conditioning protocol reflect peripheral changes that may lead to auditory central adaptations, CR levels were evaluated in the CN, the first relay structure in the central auditory pathway (Figures 8A, 9A). In control rats, CR immunostaining was observed within neuronal cell bodies that were darkly immunostained and embedded in a less stained neuropil throughout the rostrocaudal extent of the AVCN and PVCN (arrows in Figures 8B, 9B). As described above for the spiral ganglion and the spiral ligament, increases in CR levels in both neuronal cell bodies and neuropil were observed at 24 h after the first session of sound conditioning (1S1H24H; arrows in Figures 8C, 9C). The immunostaining continued elevated in 1S1H72H (arrows in Figures 8D, 9D), 4S1H24H (arrows in Figures 8E, 9E) and 4S1H72H (arrows in Figures 8F, 9F) conditions when compared to control animals. The statistical analysis revealed that overall mean gray level of CR immunostaining in the AVCN and PVCN was significantly higher in all experimental animals than in control rats (Figures 8G, 9G; Table 7). Moreover, the mean gray level in the 1S1H24H group was significantly the highest among all the experimental conditions (Figures 8G, 9G; Table 7). This upregulation for the AVCN ranged from 45.68 (1S1H24H) to 16.70% (4S1H72H; Figure 8H) and for the PVCN 30.72 (1S1H24H) to 17.56% (4S1H72H; Figure 9H) when compared to the mean gray level in controls.

**Table 7:** **Mean gray level of CR immunostaining in the AVCN and PVCN in control and experimental animals**.

		**AVCN**	**PVCN**
	Control (1)	134.87 ± 7.42	142.71 ± 5.07
	1S1H24H (2)	196.47 ± 5.17	186.55 ± 7.03
	1S1H72H (3)	172.27 ± 2.91	173.66 ± 5.77
	4S1H24H (4)	162.76 ± 1.49	168.38 ± 3.71
	4S1H72H (5)	157.39 ± 2.33	167.78 ± 3.24
	ANOVA	*F*_(4, 28)_ = 23.94 ^***^	*F*_(4, 28)_ = 9.11 ^***^
		**Significance levels**	
Statistical comparison	1 vs. 2	^***^	^***^
	1 vs. 3	^***^	^**^
	1 vs. 4	^*^	^*^
	1 vs. 5	^*^	^*^
	2 vs. 3	^*^	NS
	2 vs. 4	^**^	^*^
	2 vs. 5	^***^	^*^
	3 vs. 4	NS	NS
	3 vs. 5	NS	NS
	4 vs. 5	NS	NS

*Values are means ± standard errors*.

* *p < 0.05*;

** *p < 0.01*;

*** *p < 0.001*;

*NS, No significant*.

#### nNOS immunostaining in the AVCN and PVCN

**Figure 10:**
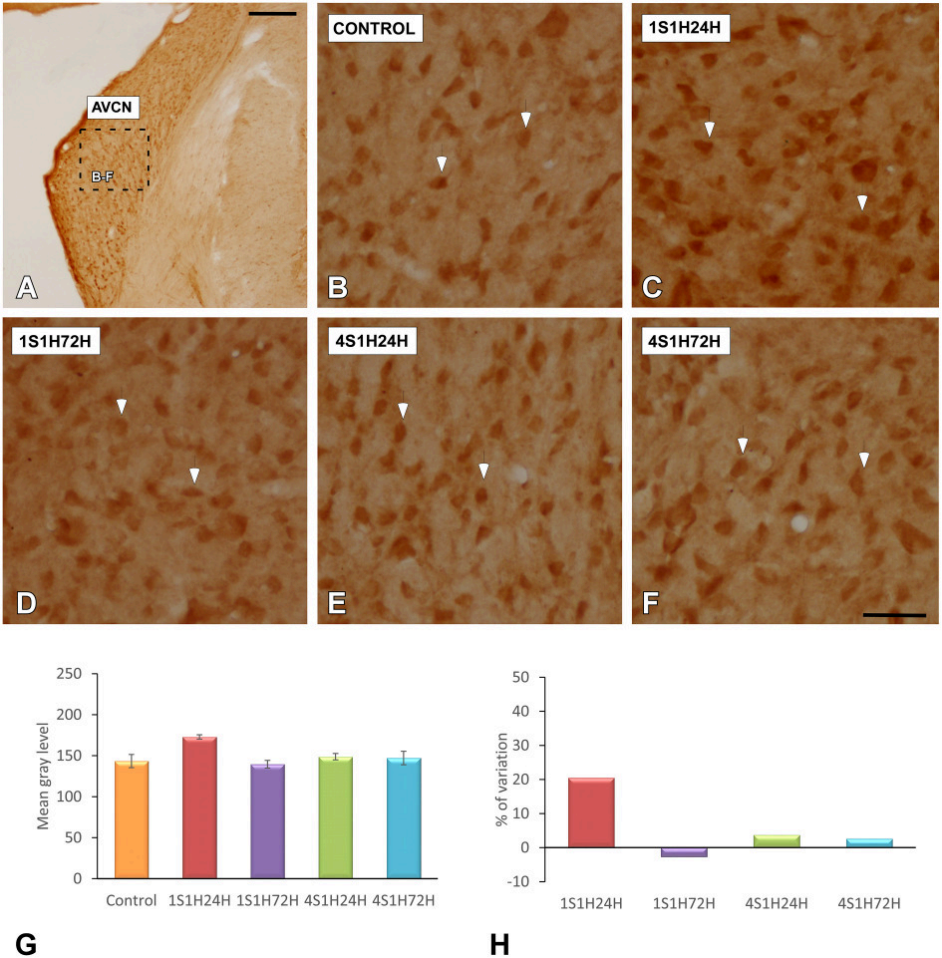
**Digitized images illustrating nNOS immunostaining in the AVCN in control and experimental Wistar rats**. nNOS-immunostaining was increased by 24 h after the first session (arrows in **C**), and diminished in 1S1H72H (arrows in **D**), 4S1H24H (arrows in **E**), and 4S1H72H (arrows in **F**) conditions to reach normal values (arrows in **B**). Bar graphs indicate the mean gray level of nNOS immunostaining in the AVCN **(G)** and its percentage of variation **(H)** in the experimental conditions relative to control. The square box in **(A)** indicates the approximate location of the high-magnification images shown in **(B,C,E,F)**. Scale bars represent 250 μm in **(A)**, and 50 μm in **(F)**.

**Figure 11:**
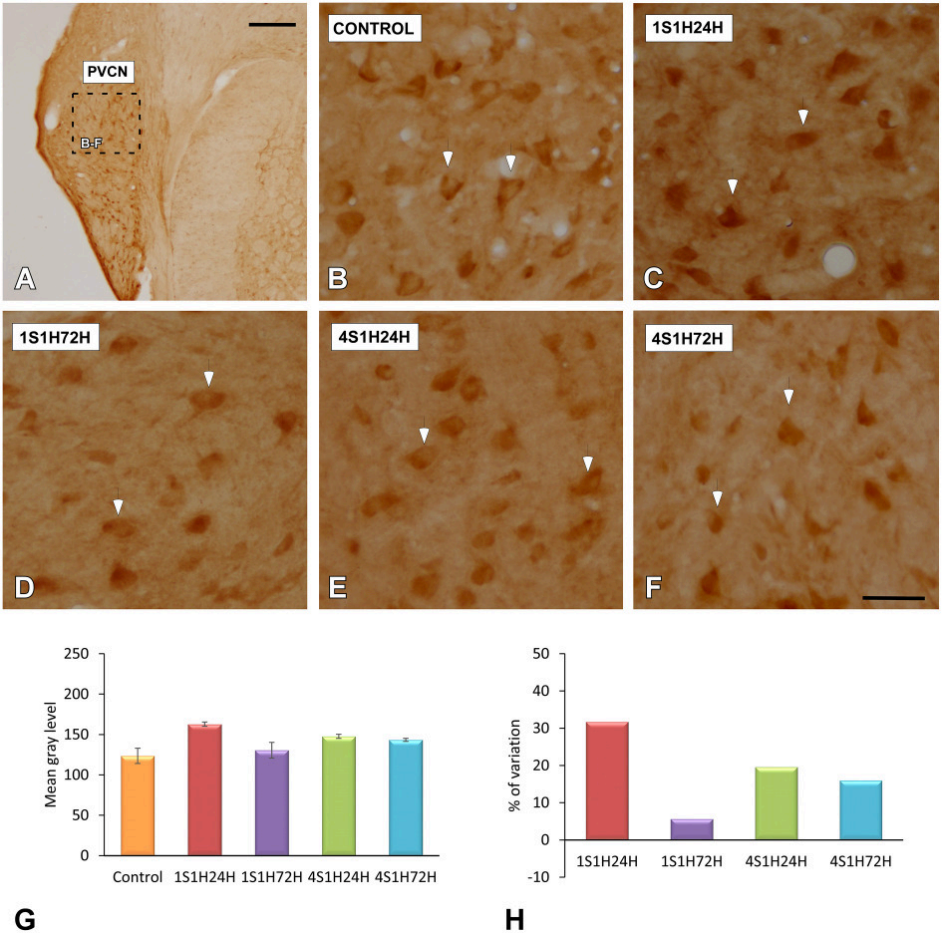
**Digitized images illustrating nNOS immunostaining in the PVCN in control and experimental Wistar rats**. Similar to that in the AVCN, nNOS immunostaining in the 1S1H24H condition was increased (arrows in **C**) when compared to the control condition. Although nNOS levels decreased in 1S1H72H (arrows in **D**) rats, they increased slightly in 4S1H24H (arrows in **E**) and 4S1H72H (arrows in **F**) animals when compared to the control condition (arrows in **B**). Bar graphs indicate the mean gray level of nNOS immunostaining **(G)** and the percentage of variation **(H)** in the experimental conditions with respect to control. The square box in A indicates the approximate location of the high-magnification images shown in **(B,C,E,F)**. Scale bars represent 250 μm in **(A)**, and 50 μm in **(F)**.

To determine whether upregulation of nNOS-immunostaining in the cochlea results in parallel modifications at the central level, changes in nNOS levels were evaluated in the CN as well (Figures 10A, 11A). nNOS immunostaining had a similar distribution pattern in both subdivisions of the CN. In control rats, the immunostaining was located mostly within CN neurons that were moderately immunostained (Figures 10B, 11B). In the AVCN (Figure 10C) and PVCN (Figure 11C) the immunostaining was highest 24 h following the first noise exposure session (1S1H24H) and decreased in both nuclei in 1S1H72H rats (Figures 10D, 11D). During the fourth session, nNOS levels in the AVCN were similar to control in 4S1H24H (Figure 10E) and 4S1H72H (Figure 10F) groups (Figures 11E,F), while remaining elevated in the PVCN (Figure 11E for 4S1H24H and Figure 11F for 4S1H72H). Determination gray levels demonstrated that in fact the mean gray level in the 1S1H24H was significantly higher than that in control and in the other experimental groups in the AVCN and PCVN (Figures 10G, 11G, Table 8). No other differences were detected among animals in the AVCN, indicating that nNOS immunostaining returned to the normal values by 72 h after the first session of noise conditioning. However, in the PCVN the mean gray levels in 4S1H24H and 4S1H72H rats were significantly higher than in control animals. The nNOS levels in the AVCN for the 1S1H24H group relative to control animals were 20.85%, while the values for the 1S1H72H, 4S1H24H, and 4S1H72H rats ranged from −2.68 to 3.74% (Figure 10H). The percentage of variation of the immunostaining in the PVCN of exposed animal varied from 31.71 to 5.63% (Figure 11H).

**Table 8:** **Mean gray level of NOS immunostaining in the AVCN and PVCN in control and experimental animals**.

		**AVCN**	**PVCN**
	Control (1)	143.45 ± 8.01	123.59 ± 9.41
	1S1H24H (2)	172.87 ± 2.68	162.78 ± 2.65
	1S1H72H (3)	139.60 ± 4.69	130.55 ± 9.67
	4S1H24H (4)	148.81 ± 3.86	147.73 ± 2.49
	4S1H72H (5)	147.14 ± 8.17	143.32 ± 1.92
	ANOVA	*F*_(4, 32)_ = 7.05 ^***^	*F*_(4, 32)_ = 6.68 ^**^
		**Significance levels**	
Statistical comparison	1 vs. 2	^**^	^**^
	1 vs. 3	NS	NS
	1 vs. 4	NS	^*^
	1 vs. 5	NS	^*^
	2 vs. 3	^**^	^*^
	2 vs. 4	^*^	^*^
	2 vs. 5	^*^	^*^
	3 vs. 4	NS	^*^
	3 vs. 5	NS	^*^
	4 vs. 5	NS	NS

*Values are means ± standard errors*.

* *p < 0.05*;

** *p < 0.01*;

*** *p < 0.001*;

*NS, No significant*.

#### SYN immunostaining in the CN

**Figure 12:**
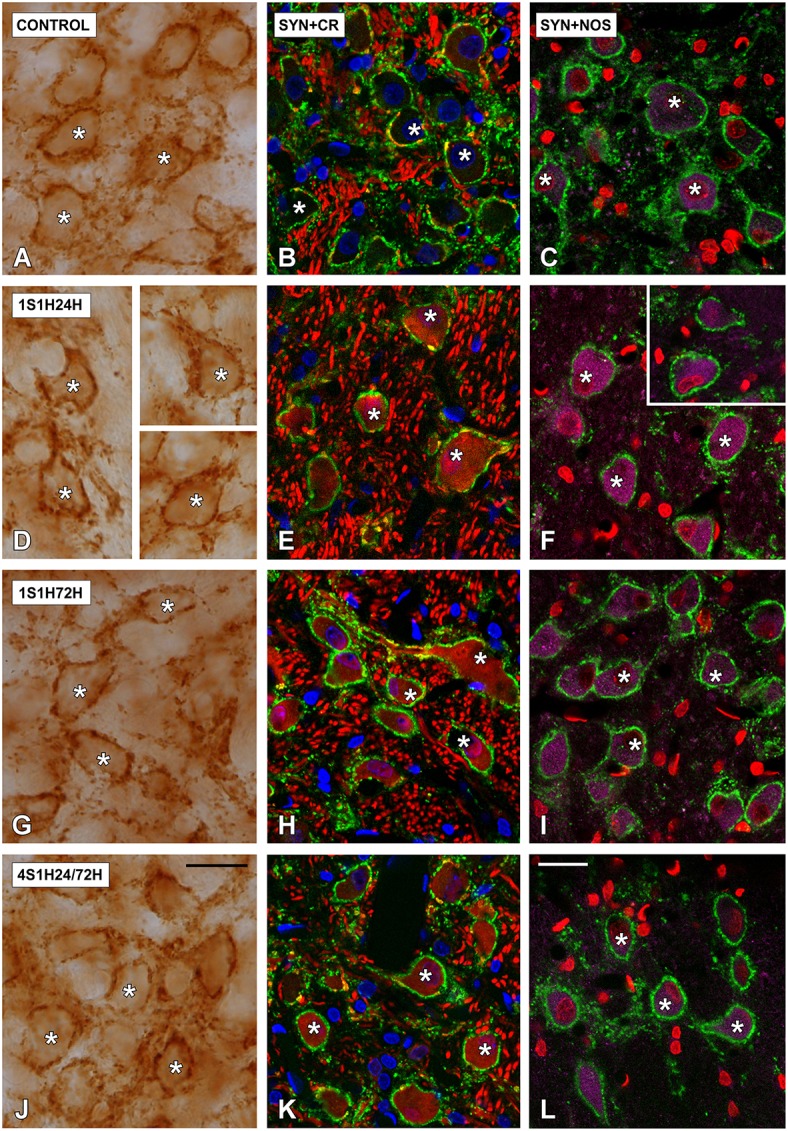
**Digitized high magnification images depicting SYN immunostaining in the AVCN in control and noise-exposed rats**. The staining consisted of punctate deposits in the neuropil and perisomatic profiles that were seen surrounding unstained cochlear nucleus neurons (asterisks in **A,D,G,J**) immunostained either with CR (asterisks in **B,E,H,K**) or with NOS (asterisks in **C,F,I,L**). Although there were no differences in the distribution pattern of SYN among groups, significant increases in CR and NOS staining were evident in 1S1H24H **(E,F)** rats when compared to control **(B,C)**. Scale bars represent 25 μm in **(J,L)**.

**Figure 13:**
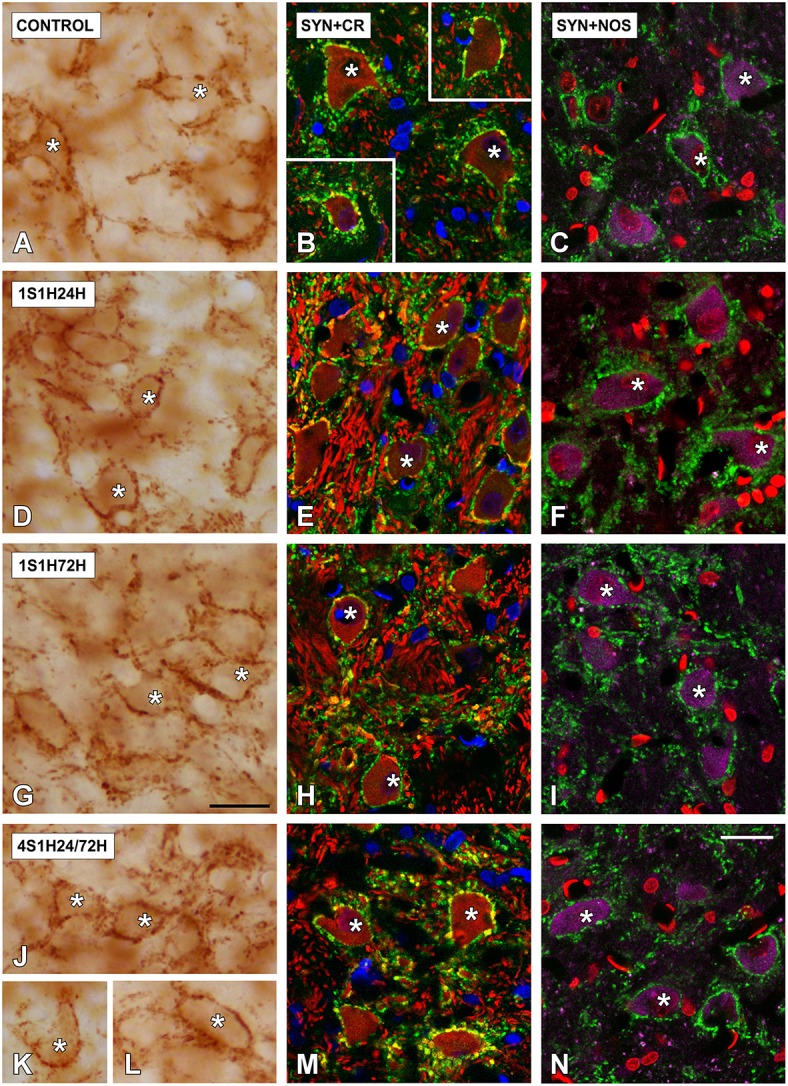
**Digitized high magnification images depicting SYN immunostaining in the PVCN in control and noise-exposed rats**. Stained endings mainly distributed around either CR or NOS immunostained cochlear nucleus neurons (asterisks in **A–N**). The distribution pattern of SYN immunostaining was not different among groups. Note that there was an upregulation in NOS and CR staining by 24 h after the first session **(D–F)**. Scale bars represent 25 μm in **(G,N)**.

In order to determine if CR and nNOS modifications in the cochlea are related to synaptic changes in the CN, SYN immunostaining was assessed before and after the sound conditioning protocol. In the control condition, the immunostaining appeared as punctate deposits in the neuropil and perisomatic profiles surrounding unstained neurons in both AVCN (Figures 12A–C) and PCVN (Figures 13A–C) as previously described (Fuentes-Santamaría et al., 2005a, 2007a). Following the sound conditioning protocol there were no apparent modifications in the SYN immunostaining pattern in either the AVCN (Figures 12D–L) or the PVCN (Figures 13D–N) at any of the survival times evaluated when compared to the control animals. Statistical analysis of the gray level measurement of SYN demonstrated that there was no significant interaction between the time post-exposure and the mean gray level of the SYN immunostaining either in the AVCN (Table 9) or in the PCVN (Table 9) when compared to experimental and control rats or when the experimental groups were compared among them. The variations of the mean gray level in the experimental animals compared to the control condition ranged from 8.16 to 3.16% for the AVCN, and from −1.73 to 5.09% for the PVCN. Additionally, no significant effects of the time post-exposure and the area of SYN immunostaining were detected when the values of the exposed animals were compared to those in control rats in either the AVCN (Table 9) or the PVCN (Table 9). The immunostained area after the sound exposure varied from −3.72 to 1.01% for the AVCN and from −2.16 to 2.61% for the PVCN.

**Table 9:** **Synaptophysin immunostaining in the AVCN and PVCN in control and experimental animals**.

		**AVCN**			**PVCN**	
		**MGL**	**ISA (μm^2^)**		**MGL**	**ISA (μm^2^)**
Control (1)		128.24 ± 5.65	1591.66 ± 43.18		128.58 ± 8.12	1551.49 ± 46.89
1S1H24H (2)		134.94 ± 3.98	1596.12 ± 31.50		121.44 ± 4.32	1585.68 ± 56.07
1S1H72H (3)		138.70 ± 4.10	1537.89 ± 61.26		129.87 ± 3.93	1518.05 ± 43.91
4S1H24H (4)		132.42 ± 4.67	1607.81 ± 21.91		124.40 ± 9.97	1535.04 ± 61.16
4S1H72H (5)		133.30 ± 4.98	1532.48 ± 61.66		127.88 ± 3.39	1592.06 ± 50.73
ANOVA		*F*_(4, 25)_ = 0.67 NS	*F*_(4, 25)_ = 0.23 NS		*F*_(4, 25)_ = 0.61 NS	*F*_(4, 25)_ = 0.36 NS
		**Significance levels**				
Statistical comparison	1 vs. 2	NS	NS	NS		NS
	1 vs. 3	NS	NS	NS		NS
	1 vs. 4	NS	NS	NS		NS
	1 vs. 5	NS	NS	NS		NS
	2 vs. 3	NS	NS	NS		NS
	2 vs. 4	NS	NS	NS		NS
	2 vs. 5	NS	NS	NS		NS
	3 vs. 4	NS	NS	NS		NS
	3 vs. 5	NS	NS	NS		NS
	4 vs. 5	NS	NS	NS		NS

*Values are means ± standard errors. MGL, Mean gray level of synaptophysin immunostaining; ISA, Immunostained area of synaptophysin. NS, No significant*.

## Discussion

The findings in the present study demonstrate that following exposure to a non-damaging repetitive sound conditioning protocol in Wistar rats there is a toughening effect expressed as a smaller threshold shift, faster recovery of the threshold shift, and a reduction of noise exposure effect on ABR wave amplitudes and latencies. This phenomenon initiates in the cochlea and propagates to brainstem auditory nuclei, as demonstrated by an upregulation of CR and nNOS levels in both peripheral (cochlea) and central auditory structures such as the CN of noise-exposed rats. The time course of increase in CR and nNOS levels were coincident with functional alterations in the ABR recordings during the sound conditioning protocol. These results lead to the suggestion that “toughening” the auditory system activates protective mechanisms to minimize cell injury resulting from subsequent noise exposures which might be related in part by activation of CR and nNOS signaling pathways.

It is well-known that noise overexposure is the major preventable cause of acquired permanent hearing impairment, with more than 500 million people in the world at risk of developing noise-induced hearing loss (NIHL; Sliwinska-Kowalska and Davis, 2012; Basner et al., 2014). One of the effects of noise overexposure is irreversible damage and/or death of sensory hair cells in the Organ of Corti that in turn lead to a PTS (Clark and Bohne, 1999; Daniel, 2007; Sliwinska-Kowalska and Davis, 2012). Depending on the age at which NIHL occurs, this condition might be associated with depression and low self-esteem, frustration, social withdrawal and, in general, cognitive decline that may lead to profound alterations on the individual's life quality, with a concomitant significant impact on public health care (Clark and Bohne, 1999; Daniel, 2007; Huang and Tang, 2010; Ciorba et al., 2012; Kidd and Bao, 2012; Sliwinska-Kowalska and Davis, 2012; Basner et al., 2014). However, noise overexposure also can result in a completely different outcome: non-damaging noise-induced priming, which reduces the auditory threshold shift and pathology that follows a noise exposure (Niu and Canlon, 2002a,b).

Wistar rats are appropriate animal models for the study of noise-induced toughening (Pukkila et al., 1997). The present results in Wistar rats corroborate earlier work showing that there is a progressive reduction in the noise-induced threshold shift following a series of non-damaging noise exposures (Pukkila et al., 1997; Ahroon and Hamernik, 1999, 2000; Attanasio et al., 1999; Henderson et al., 1999; Hamernik and Ahroon, 1999b; Gourévitch et al., 2009). The threshold shift observed at the beginning of the interrupted repetitive sound conditioning protocol was not present at the end of the noise stimulation (Pukkila et al., 1997). Our data show that the protective toughening effect is also reflected in the amplitudes and latencies of the evoked ABR waveforms as well. Wave amplitudes were reduced or even negligible, and the time latencies, at medium and higher frequencies, were significantly longer following the first noise exposure relative to control measurements; and these effects were significantly reduced or absent following the 4th noise exposure. This improvement in ABR auditory thresholds, wave amplitudes and wave latencies, during the noise-induced toughening indicate that toughening reflects intrinsic cochlear adaptive mechanisms enhancing peripheral and central neuronal responsiveness after successive noise exposures. Thus, auditory neurons respond effectively to the auditory stimulus (normal thresholds), in adequate number (normal wave amplitudes) and precise time (normal wave latencies; Gourévitch et al., 2009), as it occurs in normal conditions.

The current study shows that at the beginning of the non-damaging noise-induced toughening protocol there were significant increases in CR immunostaining in the spiral ganglion and the spiral ligament. Similar increases in the calcium-binding protein calmodulin have also been observed in the Organ of Corti of the guinea pig conditioned cochlea (Zuo et al., 2008). Although CR levels were slightly decreased at the end of the stimulation protocol, they remained significantly increased relative to the CR levels observed in unexposed rats. Importantly, increases in CR levels were also detected in the central auditory system. CR upregulation was observed in both the AVCN and the PVCN at 24 h after de first session of the noise-induced toughening protocol and persisted through the last session. These findings of a simultaneous upregulation in CR levels in peripheral and central pathways during the toughening effect in Wistar rats may reflect increased intracellular Ca^2+^ levels produced during the sound conditioning protocol (Zuo et al., 2008). Although beyond the scope of this paper, it is important to note that future work would be needed to fully quantify the proportion of labeled/unlabeled CR cells for each condition.

Noise-induced Ca^2+^ upregulation has been found following sound exposure protocols that produce either TTS or PTS (Fridberger et al., 1998; Jacob et al., 2013). During noise overstimulation, an increase in intracellular Ca^2+^ concentration is observed in both sensory hair cells and supporting cells (Jacob et al., 2013). Such an increase has been shown to alter the Organ of Corti stiffness (Jacob et al., 2013) and contraction abilities (Fridberger et al., 1998), leading to a transient or permanent alteration in auditory function (Fridberger et al., 1998; Jacob et al., 2013). A similar noise-induced Ca^2+^ upregulation also has been described in the CN following noise overexposure that leads to TTS or PTS (Gröschel et al., 2011). Under normal conditions, Ca^2+^ is involved in regulating several processes in the cochlea including the force generated by the hair bundle and length of outer hair cells (Jacob et al., 2013), firing of spiral ganglion neurons (Lv et al., 2012; Davis and Crozier, 2015), and blood flow and potassium homeostasis through the spiral ligament fibrocytes (Liang et al., 2003; Dai and Shi, 2011). This activity-dependent ion also regulates critical neuronal functions in central auditory nuclei including synaptic plasticity, neurotransmission and cellular viability (Ghosh and Greenberg, 1995; Zirpel et al., 1998, 2000; Förster and Illing, 2000; Stack and Code, 2000; Zettel et al., 2001). Considering that auditory neurons have one of the highest activity rates among neurons in the nervous system, their proper function critically depends on Ca^2+^ homeostasis (Parks et al., 1997; Hack et al., 2000). As calcium-binding proteins are critical for the maintenance of intracellular Ca^2+^ levels (Baimbridge et al., 1992), increased levels of these proteins during the noise-induced protocol may regulate Ca^2+^ concentration, which might be essential to achieve the toughening effect.

NO, is another signaling molecule regulated by cochlear afferent activity which, depending on its concentration, will participate in key physiological processes such as neuronal transmission, endolymph homeostasis and blood flow regulation (Heinrich and Helling, 2012) or in pathological events including cytotoxicity and dysregulated cell death, with impact in hearing disorders (Heinrich and Helling, 2012). Noise-induced TTS in guinea pigs produces NO upregulation in the stria vascularis and the spiral ligament along with increased auditory thresholds (Chen et al., 2005; Heinrich and Helling, 2012). While auditory thresholds returned to normal values 2 days after the noise overexposure, elevated NO levels persisted for 5 more days (Chen et al., 2005; Heinrich and Helling, 2012). Consistent with this view, we observed that during the toughening effect there was an upregulation in nNOS-immunostaining in peripheral and central structures of exposed animals relative to unexposed rats. The time-course of this upregulation was similar to that described for CR, increasing by 24 h after the first session of stimulation and, remaining slightly higher, at least for the PVCN, than in control animals following the last session of the noise-induced protocol. These findings indicate that the expression and distribution of NOS in auditory nuclei is temporally and spatially correlated with CR and suggest a possible role of both molecules during the noise-induced toughening effect.

Multiple studies have established that NO is generated by three NOS isoforms and two of its constitutive isoforms, nNOS and eNOS, are Ca^2+^/calmodulin dependent (Chen et al., 2005; Lin et al., 2011; Förstermann and Sessa, 2012; Heinrich and Helling, 2012). Thus, we propose that activity-dependent elevations in Ca^2+^ concentration, occurring during the noise-induced priming, induce an increase in NO levels in cochlear structures. Although, it seems evident that CR plays a key role in buffering Ca^2+^during the toughening effect, the mechanism by which NO contributes to it is unknown. Nevertheless, there is consensus that excess NO levels that follows an increase in Ca^2+^ concentration has a deleterious effect in the cochlea, likely because of the contribution of NO, as a free radical, to oxidative stress and subsequent increase in auditory thresholds (Ruan, 2002; Chen et al., 2005; Heinrich and Helling, 2012). However, it has been demonstrated that under conditions favoring relative ischemia, such as those induced by noise overstimulation, NO could exert a protective effect (Ruan, 2002; Chen et al., 2005). Consistent with this idea, the present data also suggest that during the toughening process NO may have dual roles in auditory nuclei, deleterious at the beginning and protective at the end of the noise protocol. Thus, during the first session of the noise-induced toughening protocol, increases in neuronal activity induce an increase in intracellular Ca^2+^ concentration activating successively eNOS and nNOS signaling. Such activation would result in an excess of NO concentration that contributes to the auditory threshold shift. These observations are consistent with previous studies in which noise overstimulation induces upregulation in NO levels that have been shown to increase auditory thresholds (Ruan, 2002; Chen et al., 2005; Heinrich and Helling, 2012). The subsequent increase in CR levels diminish Ca^2+^ concentration and therefore may reduce NO levels. NO reduction below deleterious levels may contribute to reduce the threshold shift to control values at the end of the conditioned protocol. However, NO levels still above normal may increase cochlear blood flow and reduce ischemia that occurs during the noise overstimulation (Ruan, 2002; Chen et al., 2005; Heinrich and Helling, 2012). Although, more studies are needed to elucidate whether activation of CR and NOS signaling pathways are instrumental in the protection of the auditory system provided by noise-induced toughening/conditioning, our findings suggest that the activity-dependent interplay between CR and NO in peripheral and central auditory structures may play a role in the genesis and/or maintenance of the noise-induced toughening effect.

Changes in cochlear activity have been associated with transynaptic changes in the morphology and/or biochemistry of auditory nerve and central auditory synapses (Benson et al., 1997; Alvarado et al., 2004, 2007a; Fuentes-Santamaría et al., 2005a). Previous studies in young and adult ferrets have demonstrated that in response to bilateral deafness there are plastic adaptations in central auditory nuclei, which are reflected as changes in neuronal excitability, modifications in calretinin immunostaining, and synaptic remodeling (Alvarado et al., 2004, 2007a; Fuentes-Santamaría et al., 2005a, 2007a). Since increased levels of the synaptic vesicular protein SYN have been associated with an upregulation in either the number of vesicles contained in each terminal or in the number of synaptic endings (Russell and Moore, 2002; Alvarado et al., 2004), we used this marker to assess synaptic plasticity in the cochlear nucleus induced by the sound conditioning protocol. Our CR and NOS immunostaining results demonstrate the effect of the sound conditioning toughening is not restricted to the inner ear, but also drives changes in the CN. Such upregulation occurs without significant changes in presynaptic terminals, as no modifications in the mean gray level or the immunostained area of SYN-immunostaining were observed in the CN. However, it is important to note that we cannot rule out the possibility that postsynaptic modifications such as increases/decreases in the number or efficacy of glutamate receptors, and /or changes in their channel properties/kinetics may also occur in the CN following interrupted noise exposure.

In conclusion, the present results clearly demonstrate that following a non-damaging interrupted repetitive sound conditioning protocol there is a noise-induced toughening effect in Wistar rats which is evidenced by a reduced sensitivity to noise-induced damage, as reflected by an improvement in the auditory thresholds and in the ABR wave amplitudes and latencies. The temporal coincidence between functional and biochemical changes in peripheral (cochlea) and central (CN) auditory pathways strongly suggests that the nature of this phenomenon appears to be related, at least in part, by an activity-dependent relationship between CR and nNOS. Additionally, as sound-conditioning has also been described in humans (Miyakita et al., 1992; Niu and Canlon, 2002a,b; Brashears et al., 2003), our findings also support the idea that Wistar rats are excellent and reliable animal models to evaluate the underlying mechanisms of the noise-induced priming. Understanding the protective mechanisms involved in toughening effect could help to developing therapeutic strategies that may reduce the impact of noise-induced hearing loss.

## Author contributions

All authors had full access to all the data in the study and take responsibility for the integrity of the data and the accuracy of the data analysis. Study concept and design: JA and VF. Acquisition of data: JA, VF, MG, and TJ. Statistical analysis and interpretation of data: JA and VF. Drafting of the manuscript: VF and JC. Critical revision of the manuscript for important intellectual content: JA, VF, JM, and JJ. Obtaining funding: VF, JA, and JJ.

### Conflict of interest statement

The authors declare that the research was conducted in the absence of any commercial or financial relationships that could be construed as a potential conflict of interest. The Reviewer RA declares that, despite being affiliated to the same institution as author JM, the review process was handled objectively and no conflict of interest exists.
